# Identification of Hub Genes Associated With Hepatocellular Carcinoma Using Robust Rank Aggregation Combined With Weighted Gene Co-expression Network Analysis

**DOI:** 10.3389/fgene.2020.00895

**Published:** 2020-09-30

**Authors:** Hao Song, Na Ding, Shang Li, Jianlong Liao, Aimin Xie, Youtao Yu, Chunlong Zhang, Caifang Ni

**Affiliations:** ^1^Department of Interventional Radiology, The First Affiliated Hospital of Soochow University, Suzhou, China; ^2^Department of Intervention Therapy, The Fourth Medical Center of PLA General Hospital, Beijing, China; ^3^Department of Computational Biology, College of Bioinformatics Science and Technology, Harbin Medical University, Harbin, China

**Keywords:** weighted gene co-expression network analysis (WGCNA), hub genes, hepatocellular carcinoma (HCC), biomarker, progression and prognosis

## Abstract

**Background:**

Bioinformatics provides a valuable tool to explore the molecular mechanisms underlying pathogenesis of hepatocellular carcinoma (HCC). To improve prognosis of patients, identification of robust biomarkers associated with the pathogenic pathways of HCC remains an urgent research priority.

**Methods:**

We employed the Robust Rank Aggregation method to integrate nine qualified HCC datasets from the Gene Expression Omnibus. A robust set of differentially expressed genes (DEGs) between tumor and normal tissue samples were screened. Weighted gene co-expression network analysis was applied to cluster DEGs and the key modules related to clinical traits identified. Based on network topology analysis, novel risk genes derived from key modules were mined and biological verification performed. The potential functions of these risk genes were further explored with the aid of miRNA–mRNA regulatory networks. Finally, the prognostic ability of these genes was assessed by constructing a clinical prediction model.

**Results:**

Two key modules showed significant association with clinical traits. In combination with protein–protein interaction analysis, 29 hub genes were identified. Among these genes, 19 from one module showed a pattern of upregulation in HCC and were associated with the tumor node metastasis stage, and 10 from the other module displayed the opposite trend. Survival analyses indicated that all these genes were significantly related to patient prognosis. Based on the miRNA-mRNA regulatory network, 29 genes strongly linked to tumor activity were identified. Notably, five of the novel risk genes, ABAT, DAO, PCK2, SLC27A2, and HAO1, have rarely been reported in previous studies. Gene set enrichment analysis for each gene revealed regulatory roles in proliferation and prognosis of HCC. Least absolute shrinkage and selection operator regression analysis further validated DAO, PCK2, and HAO1 as prognostic factors in an external HCC dataset.

**Conclusion:**

Analysis of multiple datasets combined with global network information presents a successful approach to uncover the complex biological mechanisms of HCC. More importantly, this novel integrated strategy facilitates identification of risk hub genes as candidate biomarkers for HCC, which could effectively guide clinical treatments.

## Introduction

Hepatocellular carcinoma (HCC) is the sixth most common malignant tumor type and the fourth leading cause of cancer-related deaths worldwide, with approximately 841,000 new cases and 782,000 deaths each year (Bray et al., 2018). Although multiple therapies have been recently developed for HCC, prognosis remains unsatisfactory due to disease progression, recurrence, and metastasis (Budhu et al., 2006). Abnormal expression of several genes is critical in tumorigenesis and development of HCC. Recent research has shown that tumor necrosis factor-α-induced protein 8 (TNFAIP8) increases HCC cell survival by blocking apoptosis, promoting greater resistance to the anticancer drugs sorafenib and regorafenib (Niture et al., 2020). High expression of ATP/GTP binding protein like 2 (AGBL2) is associated with significantly enhanced survival and proliferation of HCC cells *in vitro* and tumor growth *in vivo* (Wang L. L. et al., 2018). Although these single genes affect the phenotype of HCC, it is not known whether they constitute the hub genes. Integration of multiple datasets and network topology structures may therefore facilitate the identification of more robust biomarkers.

Owing to the substantial improvements in high-throughput gene microarray and next-generation sequencing technologies, bioinformatics analyses are increasingly applied to explore the biological characteristics of cancers. To avoid the potential large bias caused by analysis of a single dataset, many researchers have focused on analysis of multiple datasets for HCC. Recently, Li and colleagues examined the intersection of differentially expressed genes (DEGs) of three datasets (Li and Xu, 2020) and merged the multiple datasets for analysis (Li and Xu, 2020; Li et al., 2020). In the current study, we adopted the Robust Rank Aggregation (RRA) method for the analysis of multiple integrated datasets (Kolde et al., 2012).

We downloaded nine eligible microarray datasets from the Gene Expression Omnibus (GEO), which were subjected to meta-analysis to identify robust DEGs between HCC and matched normal tissues using the RRA method. Next, weighted gene co-expression network analysis (WGCNA) was performed with the DEGs to identify the most significant modules related to clinical traits of HCC. After screening the protein–protein interaction (PPI) network (Szklarczyk et al., 2015), the 29 hub genes uploaded to miRNet^1^ were screened to construct miRNA–mRNA regulatory networks and explore their potential functions. In an external test dataset from The Cancer Genome Atlas Liver Hepatocellular Carcinoma (TCGA-LIHC) collection, 28 of these hub genes were associated with the prognosis and progression of HCC. Gene set enrichment analysis (GSEA) was further performed on the independent dataset (TCGA-LIHC) to determine the potential functions of the identified hub genes. Least absolute shrinkage and selection operator (LASSO) regression was applied to construct clinical predictive models with the aim of verifying the prognostic capability of these genes in patients with HCC. In summary, integrated analysis of multiple datasets was initially conducted, followed by comprehensive screening of hub genes strongly related to HCC using a variety of efficient bioinformatics methods and verification of the results in an external dataset. Our overall findings contribute to the elucidation of the molecular mechanisms underlying pathogenesis and identification of novel prognostic biomarkers for HCC.

## Materials and Methods

### Data Sources

We downloaded nine microarray datasets from the GEO database for RRA^2^. Access numbers of the included datasets are as follows: GSE36376 (Lim et al., 2013), GSE39791 (Kim et al., 2014), GSE45114 (Wei et al., 2014), GSE57957 (Mah et al., 2014), GSE60502 (Wang et al., 2014), GSE76297 (Chaisaingmongkol et al., 2017), GSE76427 (Grinchuk et al., 2018), GSE84005, and GSE14520 (Roessler et al., 2010). Datasets were collected up to February 1, 2020, and were included based on the following criteria: (1) gene expression data from HCC and adjacent normal tissue samples were evaluated; (2) at least 15 pairs of tumor and paracancerous tissue samples were assessed; and (3) the number of genes in a single dataset was >10,000. GSE14520 contained adequate clinical information and the largest HCC sample number (471 samples) for WGCNA and LASSO regression. Detailed information on these datasets is provided in Table 1. Additionally, the TCGA-LIHC dataset containing 374 HCC and 50 normal samples was utilized as the external validation dataset and GSEA was performed.

**TABLE 1:** Details of the eight GEO datasets about HCC.

GEO	No. of samples		Platform	References
	T	N		
GSE36376	249	193	GPL10558	Lim et al., 2013
GSE39791	72	72	GPL10558	Kim et al., 2014
GSE45114	24	25	GPL5918	Wei et al., 2014
GSE57957	39	39	GPL10558	Mah et al., 2014
GSE60502	18	18	GPL96	Wang et al., 2014
GSE76297	61	58	GPL17586	Chaisaingmongkol et al., 2017
GSE76427	115	52	GPL10558	Grinchuk et al., 2018
GSE84005	38	38	GPL5175	NA
GSE14520	471	459	GPL571&GPL3921	Roessler et al., 2010

*GEO, Gene Expression Omnibus; GPL, Gene Expression Omnibus Platform; GSE, Gene Expression Omnibus Series; T, tumor samples; N, paracancerous normal samples. There is no reference information in GSE84005. NA, not available.*

### Identification of Robust DEGs

The input data of WGCNA is usually less than 5000 genes. Therefore, preliminary screening of genes is required. In addition, DEGs (tumor vs normal tissue) can better reflect the differences in biological characteristics between tumors and normal liver tissues (Sarathi and Palaniappan, 2019). We employed “limma” (R package) to normalize and analyze the differences of each dataset downloaded from the GEO (HCC and normal samples) under a false discovery rate threshold (FDR) < 0.05 (Ritchie et al., 2015). Results from each dataset were ranked according to the fold change value of each gene. Next, “RobustRankAggreg” (R package) was implemented to analyze the results of the nine datasets for the identification of robust DEGs with adjusted *P*-values < 0.05 (Kolde et al., 2012).

### Construction of the WGCNA Network and Enrichment Analysis of Key Modules

Weighted gene co-expression network analysis was used to identify modules highly correlated with clinical traits. We applied “WCGNA” (R package) to cluster all the robust DEGs identified from the GSE14520 HCC dataset with the largest sample size (471 HCC samples) and sufficient clinical information (Langfelder and Horvath, 2008). The resulting adjacency matrix was transformed into a topological overlap matrix (TOM). Differentially expressed genes were subsequently grouped into different modules based on the TOM-based dissimilarity measure. A soft-thresholding power of 7 (scale-free R = 0.90) and minimal module size of 30 were applied. The cut height was set as 0.4 to merge similar modules.

After clustering the genetic modules, key modules associated with clinicopathological variables were determined using Pearson’s correlation coefficient, including age, hepatitis B virus (HBV) activity, alanine aminotransferase (ALT) level (≤ and >50 U/L), primary tumor size (≤ and >5 cm), multinodular characteristics, cirrhosis, tumor node metastasis (TNM) stage, Barcelona Clinic Liver Cancer (BCLC) stage, Cancer of the Liver Italian Program (CLIP) stage, AFP level (≤ and >300 ng/mL), survival status, survival time (months), recurrence status, and recurrence time (months). We selected the modules that were highly correlated with clinical traits. To establish the biological functions of the key modules, R package “clusterprofiler” was applied to perform Gene Ontology (GO) and Kyoto Encyclopedia of Genes and Genomes (KEGG) analyses on individual genes. *P*-values <0.05 were indicative of significant enrichment.

### Identification of Hub Genes Based on WGNCA Combined With PPI and Construction of miRNA–mRNA Regulatory Networks

After the identification of the key modules, genes with gene significance (GS) > 0.3 and module membership (MM) > 0.8 were taken as core genes in WGCNA. Initially, the top 100 genes with high connectivity from each module were screened, of which the top 30 were marked as “hub genes in WGCNA”. Next, we uploaded the top 100 connectivity genes to the STRING^3^ database for PPI network analysis (Szklarczyk et al., 2017). The “TSV: tab separated values” file was downloaded in the “Exports” option and imported into the Cytoscape software (version 3.7.0), whereby the top 30 genes were screened as “hub genes in PPI” by “Degree” using the “cytoHubba” (Chin et al., 2014) app. GeneMANIA is a common tool for PPI network analysis and predicting the functions of preferred genes (Warde-Farley et al., 2010). The program displays genes or gene lists using bioinformatics methods, including gene co-expression, physical interactions, gene co-location, gene enrichment analysis, and website prediction. We observed the interaction types among the hub genes and visualized the gene networks with the aid of GeneMANIA. Finally, the intersecting results of both analytical methods were used to obtain hub genes, which were uploaded to miRNet^4^ to generate a miRNA–mRNA regulatory network for establishing their potential functions.

### Verification of Hub Genes

First, GEPIA2 was employed to visualize the differential expression of the hub genes between HCC and normal tissues (one-way ANOVA). Next, we used “ggpubr” (R package) to analyze the expression patterns at different TNM stages (Kruskal–Wallis test). Stage IV samples were excluded owing to a small size of less than five samples. In addition, we used the “survival” R package to perform Kaplan–Meier (K-M) survival or unique Cox regression analysis. Results were validated in the external verification dataset TCGA-LIHC.

### GSEA and LASSO

To further explore the potential functions of the genes rarely reported in HCC, we utilized the “clusterprofiler” R package to perform GSEA for each gene. In the TCGA-LIHC dataset (normalized with the “edgeR” package), 374 HCC samples were used as the gene expression matrix. Gene lists were generated according to the order of correlation with the expression of each hub gene. The C2 reference gene sets were downloaded from the Molecular Signatures Database (MSigDB)^5^. We set an adjusted *P*-value < 0.05 as the cut-off criterion. LASSO regression is widely used in the construction of clinical prediction models (Tibshirani, 1997). Next, “glmnet” (R package) was applied to verify the potential of these genes as biomarkers. GSE14520 was used as the training set and TCGA-LIHC as the test set for the LASSO regression analysis. Each cohort was divided into two groups according to the best cutoff risk score. Finally, results were visualized with K-M and ROC curves.

## Results

### Overall Study Design

**FIGURE 1:**
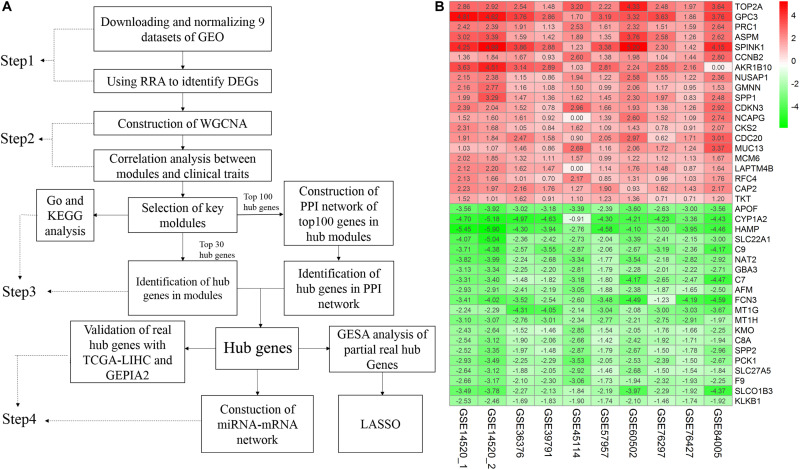
Study workflow and heatmap of the top 20 robust DEGs. **(A)** Study workflow. GEO, Gene Expression Omnibus; RRA, Robust Rank Aggregation; DEGs, differentially expressed genes; WGCNA, weighted gene co-expression network analysis; KEGG, Kyoto Encyclopedia of Genes and Genomes; GO, Gene Ontology; PPI, protein– protein interaction. GSEA, Gene Set Enrichment Analysis. GEPIA2, gene expression profiling interactive analysis. LASSO, least absolute shrinkage and selection operator **(B)** Robust DEGs analyzed by RRA. The top 20 up and down regulated genes according to the fold change value of the RRA analysis are shown in this heatmap. The row names are DEGs. The column names are GEO datasets. The numbers in each cell represent logarithmic fold change in each dataset calculated by the “limma” R package. “0” indicates that the gene corresponding to the row is missing in the data set corresponding to the column. Red indicates that DEGs are upregulated in HCC samples, while green indicates the opposite. DEGs, differentially expressed genes; GEO: Gene Expression Omnibus; RRA: robust rank aggregation. GSE14520_1, dataset from GPL571, GSE14520_2, dataset from GPL3921.

A flow chart of the study, divided into four steps, is presented in Figure 1A. Firstly, we used the RRA method to integrate and analyze the nine GEO datasets to obtain robust DEGs (Step 1). These DEGs were used to construct a WGCNA network using the GSE14520 dataset, and the key modules displaying a significant correlation with clinical traits were identified (Step 2). Hub genes were screened according to the WGCNA and PPI networks (Step 3). Finally, the hub genes were validated (Step 4).

### RRA-Based Identification of Robust DEGs Between HCC and Normal Tissues

A total of 4244 robust DEGs (2674 significantly upregulated and 1570 significantly downregulated) were identified from the nine datasets integrated using RRA (adjusted *P*-value < 0.05). As shown in Figure 1B, the 20 most significant DEGs were consistently identified among most of the datasets evaluated, signifying the robustness of the results. The majority of these genes are associated with HCC. For example, TOP2A displaying the most significant upregulation has been identified as a biomarker for HBV-related HCC (Liao et al., 2019) and APOF with the most significant downregulation is considered a tumor suppressor in HCC (Wang Y. B. et al., 2019). Significantly, AKR1B10 was not included in the GSE84005 dataset or NCAPG and LAPTM4B in the GSE45114 dataset. However, close association of these three genes with the progression of HCC has been recently reported (DiStefano and Davis, 2019; Gong et al., 2019; Wang F. et al., 2019). The RRA method effectively maximizes the retention of hub genes.

### Identification of Key Modules

**FIGURE 2:**
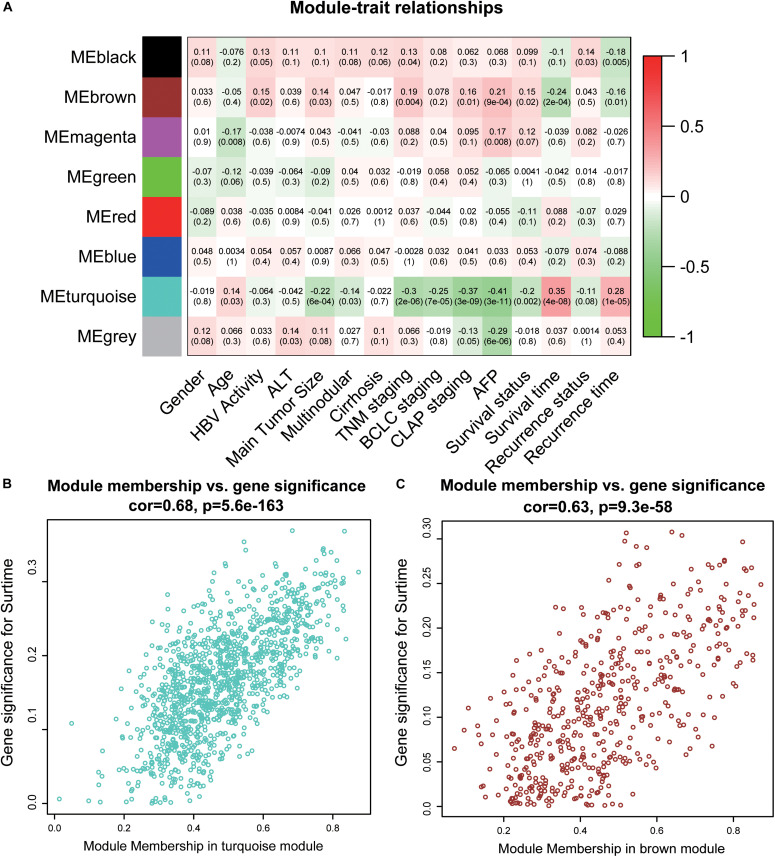
Identification of key modules. **(A)** The heatmap shows the correlation between the genes module and clinical traits of HCC. Pearson’s correlation coefficient between the gene modules and clinicopathological variables are shown, accompanied by the corresponding *P* value in brackets. Red represents positive correlation and green represents negative correlation. **(B)** The scatter plot of module eigengenes in the turquoise module. **(C)** The scatter plot of module eigengenes in the brown module. ALT, alanine aminotransferase; HBV, hepatitis B virus; TNM, tumor node metastasis; BCLC, Barcelona Clinic Liver Cancer; CLIP, Cancer of the Liver Italian Program.

To acquire the key modules, “WGCNA” (R package) was used to examine the co-expression network with the GSE14520 dataset. All DEGs derived from the RRA analysis were used as input. As shown in Supplementary Figure 1A, when the soft-thresholding power was 7 or 8, *R*^2^ was >0.9 (red line). Here, a power of β = 7 (scale-free *R*^2^ = 0.9) was selected as the soft-thresholding power to ensure a scale-free network. After applying threshold values, a total of eight modules were obtained for subsequent analysis (Supplementary Figures 2C,D). As determined from evaluation of module-trait relationships (Figure 2A), the brown and turquoise modules showed greater significance in relation to clinical information, compared with the other modules, in particular, main tumor size, TNM stage, and AFP level critical for prognosis of HCC patients (Han et al., 2014; Zhang et al., 2016) (Figures 2B,C).

### Functional Enrichment Analysis of Genes Within the Key Modules and Identification of Hub Genes

To clarify the functions of genes from the two modules, we performed separate GO and KEGG analyses. In the brown module, “DNA replication,” “cell cycle,” “p53 signaling pathway,” and “cellular senescence” were enriched in the KEGG pathway analysis (Supplementary Figure 2A) while in the turquoise module, “drug metabolism – cytochrome P450” and “chemical carcinogenesis” were enriched (Supplementary Figure 2B). These findings were consistent with previous studies reporting the involvement of the above functions in tumorigenesis of HCC. For example, Xie et al. (2018) showed that DNA replication is associated with tumor cell proliferation and prognosis of patients with HCC. Moreover, genetic variations in cell cycle pathway genes affect the disease-free survival of patients with HCC (Liu et al., 2017). The TP53 mutation is considered one of the molecular mechanisms of HCC pathogenesis (Hussain et al., 2007). Abnormal cellular senescence is a characteristic phenotype of various cancers (Chen S. L. et al., 2019). Cytochrome P450 is severely damaged and dysregulated in HCC (Yan et al., 2015). The collective findings validate the functional association of the key modules in this study with HCC. The significant biological process (BP), cellular component (CC), and molecular function (MF) GO terms of the two modules are presented in Supplementary Tables 1–6.

**FIGURE 3:**
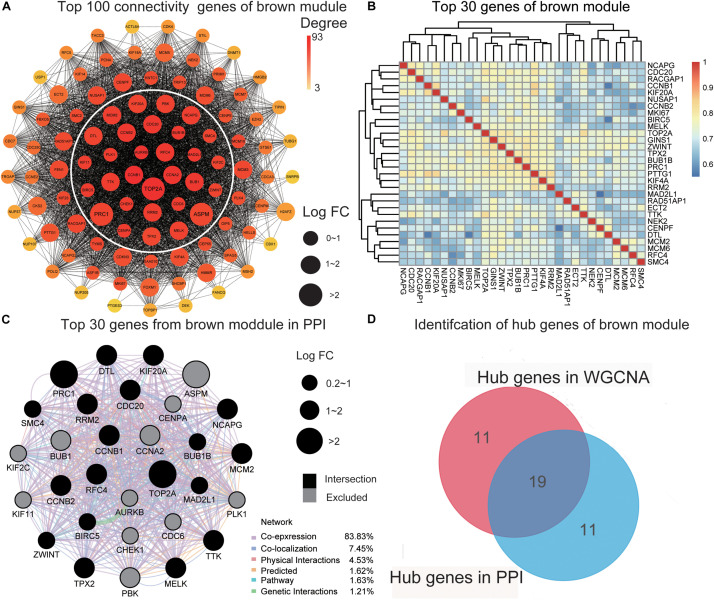
Identification of hub genes. **(A)** The PPI network of the top 100 connectivity genes from the brown module. According to degree from high to low, the gene is arranged from the center to the edge. “Hub genes in PPI” is inside the white circle. **(B)** The top 30 genes gained in WGCNA from the brown module by setting MM) > 0.8 and GS > 0.3. The correlation between these genes is shown. **(C)** The PPI network (GeneMANIA) of the top 30 genes in the brown module. Different colors of the network edge indicate the bioinformatics methods applied: physical interactions, co-expression, predicted, co-localization, pathway, genetic interactions, and shared protein domains. The size of each node indicates the value of LogFC according to the result of RRA. The black nodes present the intersection of hub genes in PPI and WCGNA. **(D)** The hub genes in the brown module are selected by PPI network and co-expression network. PPI, protein–protein interaction; MM, module membership; GS, gene significance. LogFC, log fold change; RRA, Robust Rank Aggregation. WGCNA, weighted gene co-expression network analysis.

To further screen for the most significant hub genes, we used a combination of two methods (WGCNA and PPI networks, see section “Materials and Methods”). The PPI network of the top 100 connectivity genes from the brown module is shown in Figure 3A. According to degree (high to low), the positions of genes are arranged from the inside to outside, and the top 30 considered “hub genes in PPI”. Interaction analysis of hub genes in PPI was further performed using GeneMANIA to clarify the correlations among colocalization, shared protein domains, co-expression, prediction, and pathways. As revealed by the protein–protein interaction network generated with GeneMANIA (Figure 3C), co-expression interactions accounted for the largest proportion (83.83%), consistent with the results of WGCNA. “Hub genes in WGCNA” and their correlated expression levels are shown in Figure 3B. The hub genes were obtained by selecting the intersecting results with the two methods (Figure 3D). The hub genes of the turquoise module were obtained with the same method (Supplementary Figures 3A–D). Overall, we identified a total of 29 core genes from the two key modules.

### Construction of the miRNA–mRNA Regulatory Network

**FIGURE 4:**
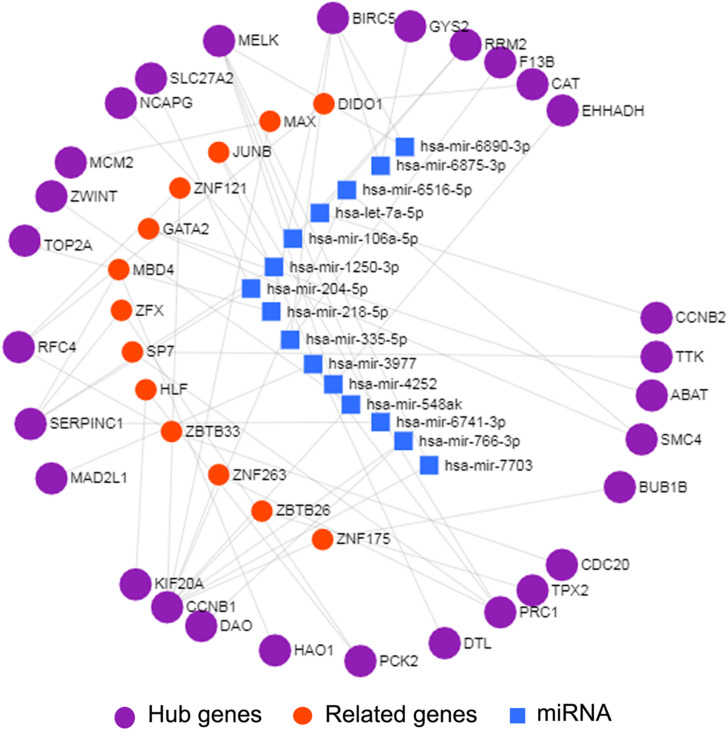
miRNA-mRNA network of 29 hub genes by miRNet. The purple spheres represent 29 hub genes; the blue squares represent the miRNA associated with the hub genes, and the red spheres represent the genes associated with the hub genes.

Interactions between miRNA and mRNA are an increasing focus of research attention. To further explore the functions of hub genes from a global perspective, a miRNA–mRNA regulatory network was constructed via miRNet (Figure 4). Previous studies suggest that a number of these miRNAs are related to HCC. For example, exosome hsa-miR-335 was identified as a therapeutic target for HCC (Wang F. et al., 2018). Furthermore, according to web-based KEGG analysis, this network is enriched in multiple tumor-related pathways (Supplementary Table 7), such as cell cycle and p53 signaling (Hussain et al., 2007; Sanchez-Vega et al., 2018; Ikeno et al., 2019). Thus our group of hub genes may play important roles in HCC through the miRNA–mRNA regulatory network.

### Verification of Hub Genes Based on the TCGA-LIHC Dataset

**FIGURE 5:**
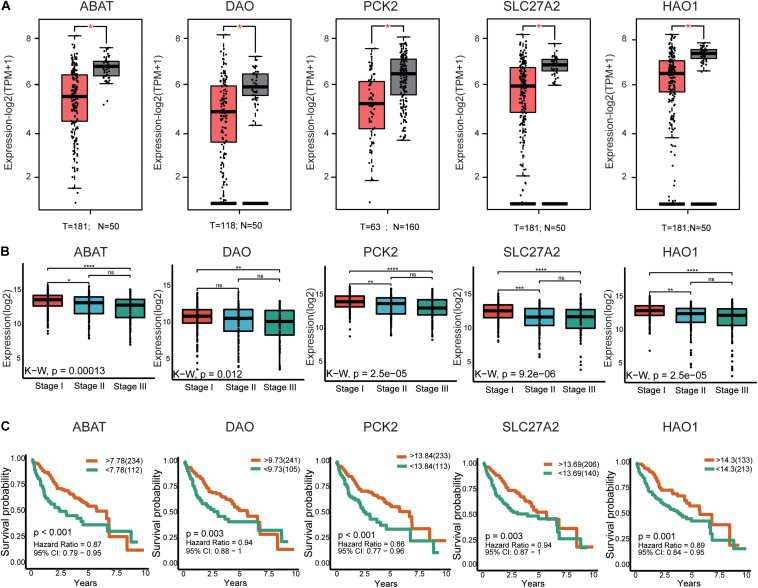
External validation of the partial hub genes in the turquoise module. **(A)** Partial hub genes, rarely reported on HCC, expression differences between HCC and adjacent normal tissues in GPEIA 2. ABAT, DAO, PCK2, SLC27A2, and HAO are downregulated in HCC tissues. “*” represents *P* value < 0.05. **(B)** Expression of ABAT, DAO, PCK2, SLC27A2, and HAO1 in HCC samples with different TNN stages. The lower the expression level of these genes indicates the more advanced stage of HCC. “*” represents *P* value < 0.05; “**” represents *P* value < 0.01; “***” represents *P* value < 0.001; “****” represents *P* value < 0.0001. **(C)** The association between ABAT, DAO, PCK2, SLC27A2, and HAO1 expression and overall survival time in the TCGA-LIHC dataset. The yellow line indicates high expression groups and the green line represents the low expression group. *T*, number of HCC samples, *N*, number of normal samples.

In total, 29 hub genes were obtained. Interestingly, TOP2A was consistently ranked first. Ten of the genes were filtered from the turquoise module (Figure 3C), which were further verified in TCGA-LIHC and GEPIA2 based on three parameters: (1) differential expression (HCC sample vs paracancerous sample), (2) TMN staging, and (3) survival analysis. In terms of expression, hub genes from the turquoise module were downregulated in HCC relative to normal samples. Notably, F13B was excluded due to lack of statistical significance. These results were validated using an external dataset (Figure 5A and Supplementary Figure 4A). Additionally, genes were differentially expressed in HCC samples with different TNM stages to a significant extent. A higher expression of these genes was correlated with an earlier TNM stage (Figure 5B and Supplementary Figure 4B). Survival analysis revealed an association of low expression of these genes with poor prognosis (Figure 5C and Supplementary Table 8). Using the same method, hub genes of the brown module (Supplementary Figure 3D) were validated, which showed an opposite trend to genes of the turquoise module (Supplementary Figures 5, 6 and Supplementary Table 8). Our collective data support critical roles of 28 of the 29 hub genes in HCC.

### GSEA of Tumor Suppressor Roles of Hub Genes

The majority of the hub genes for HCC have already been reported (Supplementary Table 9). However, DAO, SLC27A2, GYS2, HAO1, and PCK2 have not been previously studied in association with HCC. To analyze their potential functions in HCC, we performed GSEA on TCGA-LIHC RNA sequencing data. As shown in Supplementary Figure 7, three gene sets associated with tumors were defined. In samples showing a significant negative correlation of HAO1 and SLC27A2 expression with HCC, “epithelial-mesenchymal transition” (EMT) and “PI3K/Akt/mTOR” were enriched. “Wnt/beta-catenin signaling” and “MYC target v1” were significantly enriched in samples showing a negative correlation of PCK2 and DAO expression with HCC. The gene set “DNA repair” was enriched in samples showing negative correlation of ABAT and SLC27A2 expression with HCC. These mechanisms are typical tumor-associated pathways. For instance, EMT is reported to coordinate the occurrence of liver fibrosis, carcinogenesis, and proliferation and invasion of HCC cells (Giannelli et al., 2016). The activation of PI3K/AKT signaling has been shown to promote EMT (Liu et al., 2018). “Wnt/beta-catenin signaling”, “MYC target v1”, and “DNA repair” are closely related to tumorigenesis and the development of HCC (Dolezal et al., 2017; Dimri and Satyanarayana, 2020; Pardini et al., 2020). Taken together, the findings clearly suggest that these genes are closely associated with the mechanisms underlying HCC cell proliferation.

### Construction of the Novel Hub Gene Signature for Survival Prediction

**FIGURE 6:**
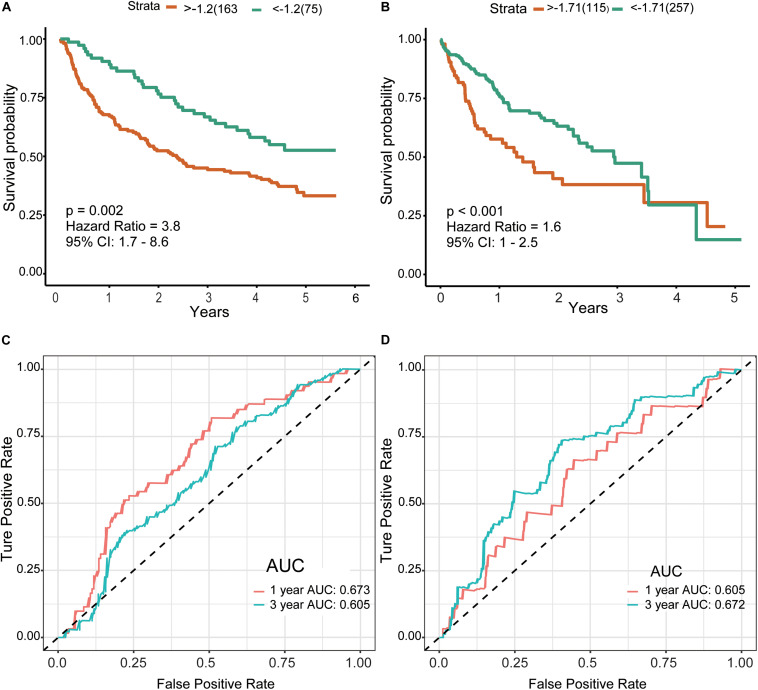
Risk score and survival analysis in training and validation datasets.**(A,B)** K–M curves for patients in GSE14520 **(A)** and TCGA-LIHC **(B)** datasets divided into high- and low-risk groups by the best cutoff values. Patients of a high risk group exhibited poorer prognosis in both cohorts. **(C,D)** ROC curves showed the predictive risk signature for patients in GSE14520 **(C)** and TCGA-LIHC **(D)** datasets on the survival rate. K–M, Kaplan–Meier; ROC, receiver operating characteristic, AUC, area under curve.

Finally, we included the above five hub genes in the LASSO regression analysis to construct a survival prediction model for HCC patients. GSE14520 was used as the training set to generate a prediction model comprising three of the genes, specifically, OSPCK2, DAO, and HAO1. The formula for calculating the prognostic risk score was as follows: (−0.0179 × expression HAO1) + (−0.0221 × expression PCK2) + (−0.1209 × expression DAO). The results of this scoring system were depicted using a K–M curve (Figures 6A,B). The high-risk group had shorter OS, both in the training (*P* = 0.002) and test (*P* < 0.001) datasets. In addition, we generated time-dependent ROC curves to evaluate the predictive effects of the three-gene signature based on the area under the curve (AUC) value. In the training cohort, one-year and three-year AUC values were 0.673 and 0.605, respectively. In the verification cohort, AUC for one year was 0.605 and that for three years was 0.672 (Figures 6C,D). Based on the results, we propose that this novel three-gene signature can serve as a reliable predictor of OS in HCC patients.

## Discussion

In this study, we used multiple bioinformatics methods to establish the biological mechanisms of HCC. To avoid the potential bias caused by DEGs in a single database, numerous studies have focused on evaluating multiple datasets (Xu et al., 2016). In the process of merging data, gene symbols that are not detected in only one dataset may be lost. For example, as shown in Figure 1B, AKR1B10, NCAPG, and LAPTM4B exist in multiple datasets and would therefore be lost if the datasets were simply merged. However, these genes are closely related to the progression of HCC (DiStefano and Davis, 2019; Gong et al., 2019; Wang F. et al., 2019). Furthermore, in dataset GSE39791, logFC values of some of the top 20 DEGs were less than 1. However, in combination with other datasets, the RRA method suggests that these genes are robust DEGs. Potential bias of results due to inclusion of only one dataset should be avoided. In the current investigation, RRA was applied to analyze nine groups of datasets to minimize bias, avoid missing hub genes, and obtain the most robust DEGs.

Weighted gene co-expression network analysis is based on the correlation between modules and clinical features, and the screening results are highly reliable and biologically meaningful (Yin et al., 2018). To our knowledge, the current study is the first to combine the RRA method with WGCNA for efficient identification of hub genes associated with HCC. Among the eight gene modules, brown and turquoise modules were closely related to clinical characteristics, such as primary tumor size, AFP level, TNM stage, and overall survival time. In addition, GO and KEGG analyses showed enrichment of both modules in multiple tumor-related pathways. For instance, DNA replication is associated with tumor cell proliferation and the prognosis of HCC (Xie et al., 2018), variations in cell cycle pathway genes affect disease-free survival of patients with HCC (Liu et al., 2017), TP53 mutation is considered one of the critical molecular mechanisms of HCC pathogenesis (Hussain et al., 2007), an abnormal cellular senescence phenotype is observed in various cancer types (Chen S. L. et al., 2019), and cytochrome P450 is severely damaged and dysregulated in HCC (Yan et al., 2015).

Next, we combined co-expression and PPI networks to screen for hub genes. After a series of strict screening steps, 29 hub genes (10 from the turquoise module and 19 from the brown module) were isolated. To explore the functions of this group of genes from the global network, miRNA–mRNA regulatory networks were generated using miRNet (Figure 4). As shown in Supplementary Table 7, specific pathways, such as cell cycle, and p53 signaling, were highlighted, both of which are closely related to tumorigenesis and the development of HCC (Hussain et al., 2007; Sanchez-Vega et al., 2018; Ikeno et al., 2019). Importantly, we used TCGA-LIHC, a dataset containing 374 HCC samples, to validate the predictive power of these hub genes in the progression and prognosis of HCC. Among the genes examined, only one (F13B) failed verification.

The involvement of the majority of these genes in HCC has been confirmed in earlier experiments (Supplementary Table 9), supporting the efficacy of our screening strategy. Among the hub genes, TOP2A, RFC, and the CCMB family have received considerable research attention. DNA topoisomerase II alpha (TOP2A) is abundantly expressed in testis, lymph node tissues, and a variety of tumor tissues, including liver cancer. Several bioinformatics analyses have validated TOP2A as a biomarker for HCC, in particular, HBV-related HCC (Liao et al., 2019). Panvichian et al. (2015) reported overexpression of TOP2A in 72.5% of tumor tissues and its significant association with the hepatitis B surface antigen (HBsAg) in serum. In addition, results of a phase III prospective randomized study showed that TOP2A is associated with the histological grade of liver cancer, microvascular invasion, early onset of malignant tumors (≤40 years), and chemotherapy resistance (Wong et al., 2009). Replication factor C subunit 4 (RFC4) has recently been identified as a hub gene affecting prognosis of patients with HCC (Kong et al., 2019). The knockdown of endogenous RFC4 suppresses HCC cell growth and enhances the chemosensitivity of HepG2 cells (Arai et al., 2009). Cyclin B1 (CCNB1) and TOP2A are considered key genes for early diagnosis of HCC (Wu et al., 2019).

Interestingly, DAO, ABAT, SL27AL, PCK2, and HAO1, all from the turquoise module, have not been shown to be associated with HCC to date, either *in vivo* or *in vitro*. However, several studies support inhibitory roles of these genes in other tumors. The peroxisomal enzyme D-amino acid oxidase (DAO) is highly expressed in the kidney, liver, and brain in mammals (Fang et al., 2008) and plays a critical role in the pathophysiology of schizophrenia (Liu et al., 2016). Earlier reports suggest that DAO inhibits glioma cell growth by inhibiting angiogenesis (El Sayed et al., 2012) and inducing apoptosis (Li et al., 2008). 4-Aminobutyrate aminotransferase (ABAT) is mainly responsible for decomposing γ-aminobutyric acid (GABA), an inhibitory neurotransmitter, into succinic semialdehyde. In basal-like breast cancer (BLBC) cells, GABA increases the intracellular Ca^2+^ concentration and effectively activates nuclear factor 1-4 (NFAT1). Consequently, ABAT expression inhibits the tumorigenicity and metastasis of BLBC cells *in vitro* and *in vivo*. Conversely, the downregulation of ABAT promotes the progression of BLBC (Chen X. et al., 2019) and resistance to endocrine therapy of inflammatory breast cancer (Jansen et al., 2015). Moreover, ABAT has been identified as a prognostic factor for renal cell carcinoma and hepatic adenocarcinoma (Reis et al., 2015; Lu et al., 2020). Chen et al. (2017) screened six genes related to HCC metastasis and prognosis through a co-expression network analysis, which led to the identification of DAO and ABAT. However, their mechanisms of action in HCC have not been clarified. Solute carrier family 27 member 2 (SLC27A2), also designated FATP2, improves the efficiency of cancer therapy by inhibiting the activity of polymorphonuclear myeloid-derived suppressor cells (PMN-MDSCs) (Veglia et al., 2019). Phosphoenolpyruvate carboxykinase 2 (PCK2) encodes a key mitochondrial enzyme for gluconeogenesis in the liver. The overexpression of PCK2 inhibits melanoma cell growth *in vitro* and prevents tumorigenesis *in vivo* (Luo et al., 2017). More recent experiments have demonstrated an association of decreased PCK2 expression with metastasis and the recurrence of osteosarcoma (Zhang et al., 2019). Upon suppression of autophagy, levels of glucose-6-phosphatase (G6Pase) and phosphoenolpyruvate carboxykinase (PEPCK, a protein encoded by PCK2) are reduced in the human HCC cell line HepG2 (Jeon et al., 2015). Hypoxia-inducible factor 1α (HIF-1α) can promote the growth of human breast tumor-repopulating cells by downregulating PCK2 (Tang et al., 2019). However, a number of studies have reported that PEPCK coordinates the regulation of central carbon metabolism to promote tumor cell growth (Montal et al., 2015). Therefore, the biological characteristics of PCK2 in HCC requires further investigation. Hydroxyacid oxidase 1 (HAO1) is expressed mainly in the liver and pancreas. An earlier genome-wide association study in Korea showed that a single nucleotide polymorphism in HAO1 is one of the risk factors for childhood acute lymphoblastic leukemia (Han et al., 2010).

In our study, GSEA consistently supported the tumor suppressor roles of these genes in multiple carcinogenic pathways in HCC datasets. Further research is warranted to establish the mechanisms of action of these genes in HCC. The collective evidence to date suggests that these genes play a suppressive roles in the biological processes of tumors. In addition, the clinical prediction model generated using a three-gene signature showed efficacy in predicting the survival of patients with HCC and the potential as a robust biomarker. Our study has some limitations, such as the fact that the nine datasets of the training set are all microarrays and lack RNA-seq datasets. The data diversity is insufficient.

## Conclusion

Systematic analysis of the genes involved in pathogenesis of HCC using a novel integrated strategy led to the identification of two risk modules and several representative hub genes. Among these, HAO1, SCL27A2, DAO, ABAT, and PCK2, rarely reported in HCC to date, were validated as novel hub genes that may serve as effective clinical diagnostic and prognostic markers as well as therapeutic targets for HCC.

## Data Availability Statement

Publicly available datasets were analyzed in this study. This data can be found here: The datasets analyzed in the current study are available in the TCGA repository (http://cancergenome.nih.gov/) and GEO (https://www. ncbi.nlm.nih.gov/geo/).

## Author Contributions

YY contributed to the project design. CN and CZ revised and verified the article. ND, SL, JL, and AX provided the bioinformatics method support and reviewed the manuscript. HS analyzed the data and wrote the article. All authors contributed to the article and approved the submitted version.

## Conflict of Interest

The authors declare that the research was conducted in the absence of any commercial or financial relationships that could be construed as a potential conflict of interest.

## Footnotes

1 https://www.mirnet.ca/

2 https://www.ncbi.nlm.nih.gov/gds/?term=

3 https://string-db.org/

4 https://www.mirnet.ca/

5 http://software.broadinstitute.org/gsea/msigdb/index.jsp

## Supplementary Material

Supplementary Figure 1 Determination of soft-thresholding power and cut height in the WGCNA. **(A)** Analysis of scale-free index (left) and mean connectivity (right) for different soft-thresholding power (β) red line indicates signed R^2 = 0.9. **(B)** When β = 7 histogram of connectivity distribution (left) and scale-free topology R^2 = 0.9 (right). **(C)** Clustering of module eigengenes. Set the cut height as 0.4 (red line) to merge similar modules. **(D)** Dendrogram of all DEGs clustered based on a dissimilarity measure (1-TOM). Each color represents a set of gene modules.

Supplementary Figure 2 The screening of hub genes in the turquoise module. **(A)** The PPI network of top 100 connectivity genes from the brown module. “Hub genes in PPI” is inside the black circle. **(B)** The top 30 hub genes gained in WGCNA from the brown module by setting MM) > 0.8 and GS > 0.3. Correlation between these genes is shown. **(C)** PPI network (GeneMANIA) of the top 30 genes in the brown module. **(D)** Selection of hub genes that occur in both the PPI network and WGCNA. PPI, protein–protein interaction; MM, module membership; GS, gene significance. WGCNA, weighted gene co-expression network analysis.

Supplementary Figure 3 KEGG analysis for the key modules. **(A)** Brown module. **(B)** Turquoise module. KEGG, Kyoto Encyclopedia of Genes and Genomes.

Supplementary Figure 4 External validation of the rest of the hub genes in the turquoise module. **(A)** The rest of the hub genes in the turquoise module expression differences between HCC and adjacent normal tissues in GPEIA2. “^∗^” represents *P* value l < 0.05. **(B)** Expression of CAT, F13B, EHHADH, GYC2, and SERPINC1 in HCC samples with different TNN stages. “^∗^” represents *P* value < 0.05; “^∗∗^” represents *P* value l < 0.01; “^∗∗∗^” represents *P* value < 0.001.

Supplementary Figure 5 External validation of hub genes in the brown module. The hub genes from the brown module expression differences between HCC and adjacent normal tissues in GPEIA2. “^∗^” represents *P* value l < 0.05.

Supplementary Figure 6 External validation of hub genes in the brown module. Expression of these genes in HCC samples with different TNN stages. “^∗^” represents *P* value < 0.05; “^∗∗^” represents *P* value l < 0.01; “^∗∗∗^” represents *P* value < 0.001.

Supplementary Figure 7 Gene sets related to cancer. Results of GSEA related to cancer in samples negatively correlated with PCK2 **(A)**, ABAT **(B)**, HAO1 **(C)**, SLC27A2 **(D)**, and DAO **(E)** expression. Highlight 3 gene sets for each gene.

Supplementary Figure 8 Risk score distribution, survival status, and heatmaps for patients in the GSE14520 **(A)** and TCGA-LIHC **(B)** datasets divided into high- and low-risk groups.

Supplementary Figure 9 Node degree distribution plot of differentially expressed genes.

Supplementary Table 1 BP of GO analysis for brown module.

Supplementary Table 2 CC of GO analysis for brown module.

Supplementary Table 3 MF of GO analysis for brown module.

Supplementary Table 4 BP of GO analysis for turquoise module.

Supplementary Table 5 CC of GO analysis for turquoise module.

Supplementary Table 6 MF of GO analysis for turquoise module.

Supplementary Table 7 Enriched function of the miRNA-mRNA network by miRNet.

Supplementary Table 8 Survival analysis for the rest of the hub genes.

Supplementary Table 9 Biological functions of the hub genes in HCC.
