# Supplementary figures and images for: Identification of Hub Genes Associated With Hepatocellular Carcinoma Using Robust Rank Aggregation Combined With Weighted Gene Co-expression Network Analysis
Source: Front Genet. 2020 Sep 30;11:895. doi: 10.3389/fgene.2020.00895 (PMC7561391; doi:10.3389/fgene.2020.00895)

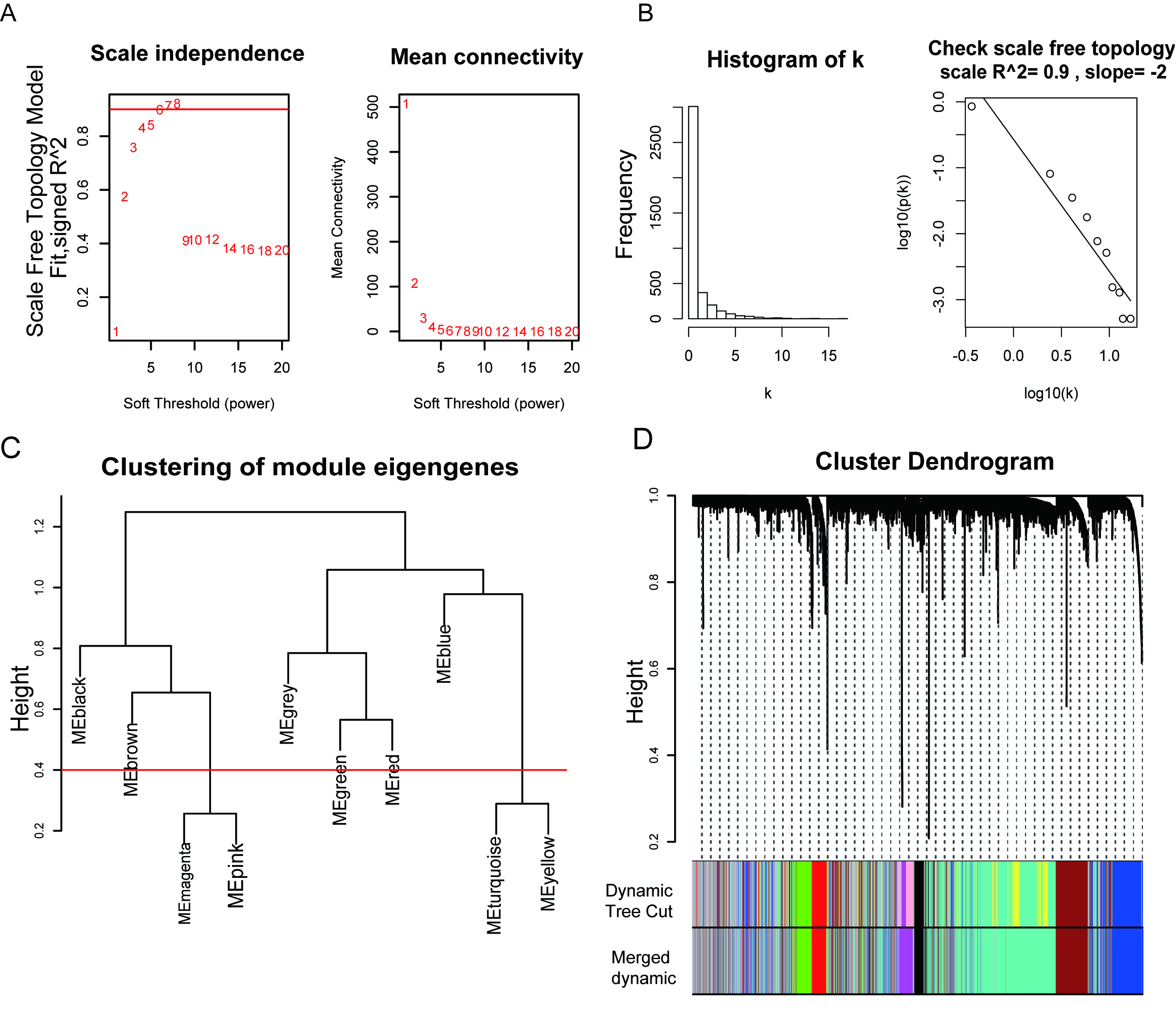

Supplement: Supplementary Figure 1 — Determination of soft-thresholding power and cut height in the WGCNA. (A) Analysis of scale-free index (left) and mean connectivity (right) for different soft-thresholding power (β) red line indicates signed R^2 = 0.9. (B) When β = 7 histogram of connectivity distribution (left) and scale-free topology R^2 = 0.9 (right). (C) Clustering of module eigengenes. Set the cut height as 0.4 (red line) to merge similar modules. (D) Dendrogram of all DEGs clustered based on a dissimilarity measure (1-TOM). Each color represents a set of gene modules. [file Image_1.TIF]

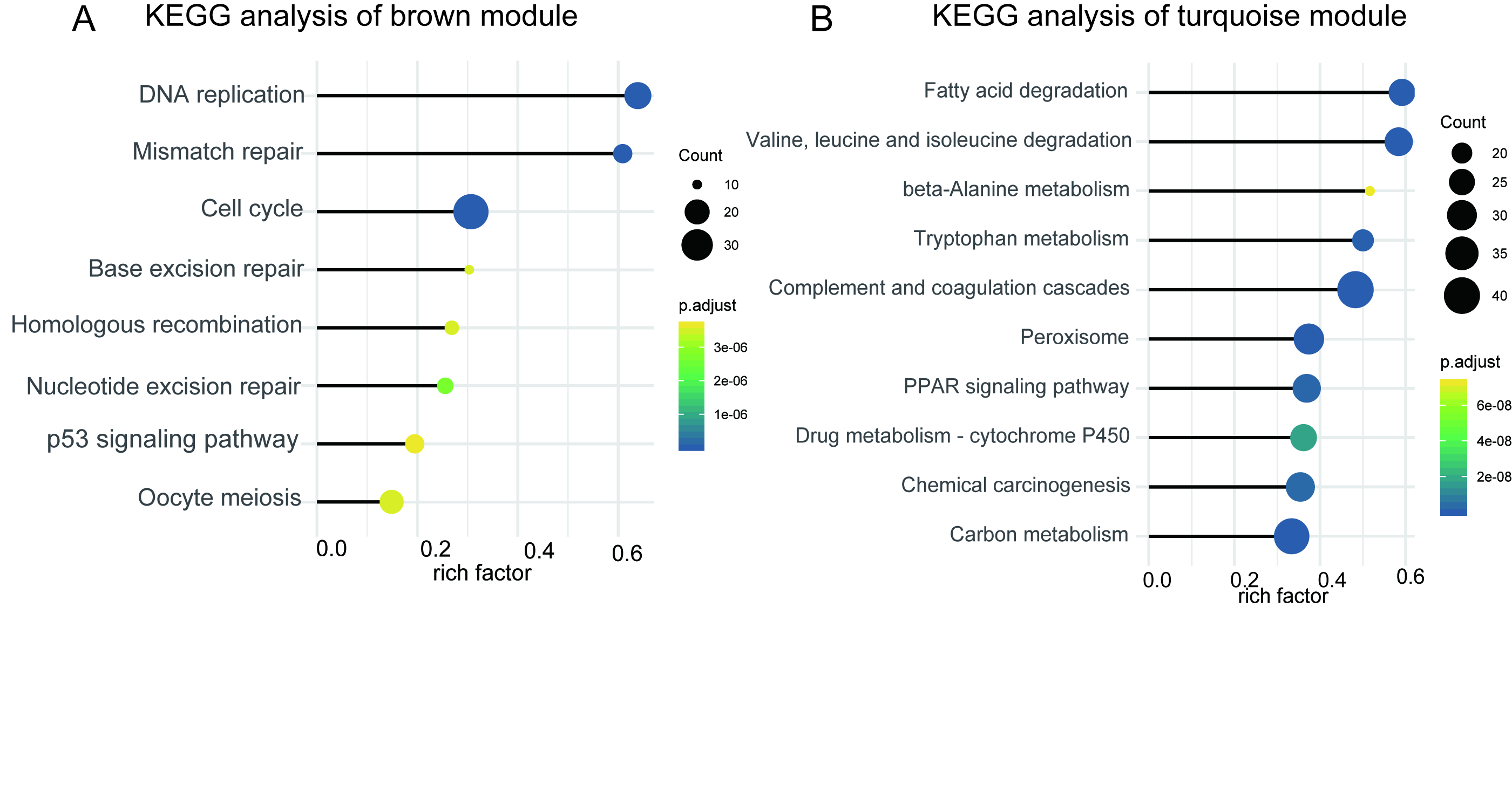

Supplement: Supplementary Figure 2 — The screening of hub genes in the turquoise module. (A) The PPI network of top 100 connectivity genes from the brown module. “Hub genes in PPI” is inside the black circle. (B) The top 30 hub genes gained in WGCNA from the brown module by setting MM) > 0.8 and GS > 0.3. Correlation between these genes is shown. (C) PPI network (GeneMANIA) of the top 30 genes in the brown module. (D) Selection of hub genes that occur in both the PPI network and WGCNA. PPI, protein–protein interaction; MM, module membership; GS, gene significance. WGCNA, weighted gene co-expression network analysis. [file Image_2.TIF]

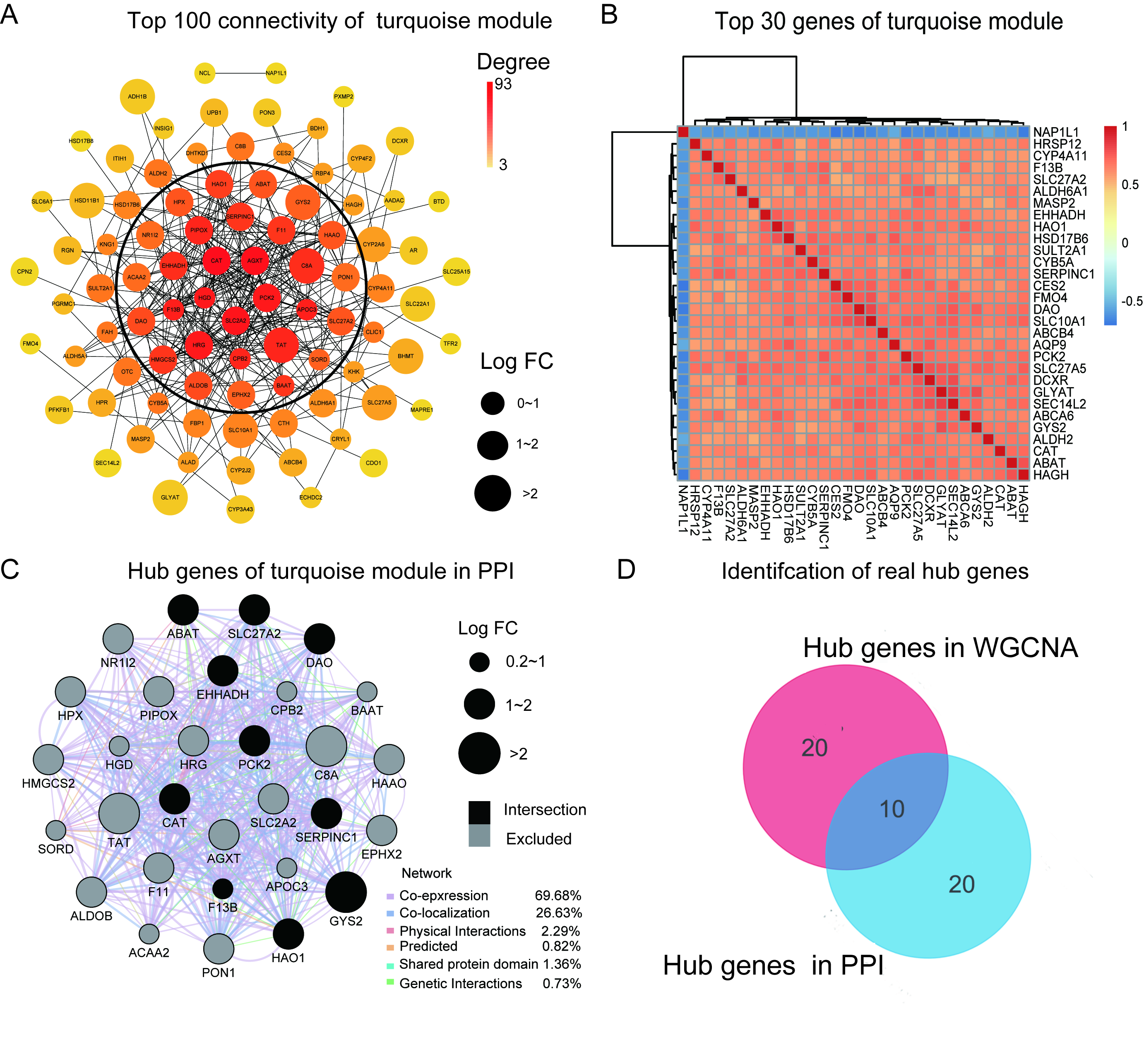

Supplement: Supplementary Figure 3 — KEGG analysis for the key modules. (A) Brown module. (B) Turquoise module. KEGG, Kyoto Encyclopedia of Genes and Genomes. [file Image_3.TIF]

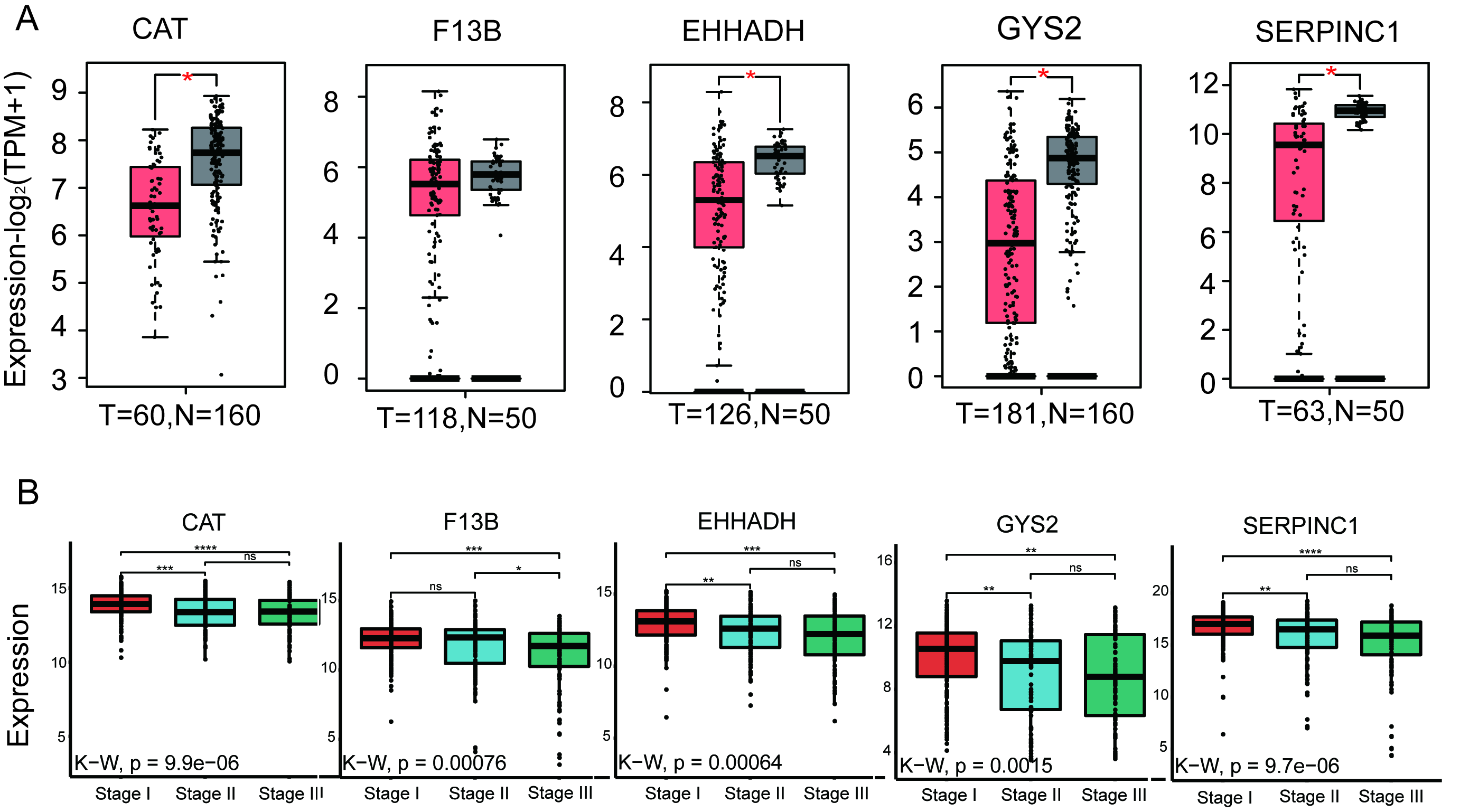

Supplement: Supplementary Figure 4 — External validation of the rest of the hub genes in the turquoise module. (A) The rest of the hub genes in the turquoise module expression differences between HCC and adjacent normal tissues in GPEIA2. “∗” represents P value l < 0.05. (B) Expression of CAT, F13B, EHHADH, GYC2, and SERPINC1 in HCC samples with different TNN stages. “∗” represents P value < 0.05; “∗∗” represents P value l < 0.01; “∗∗∗” represents P value < 0.001. [file Image_4.TIF]

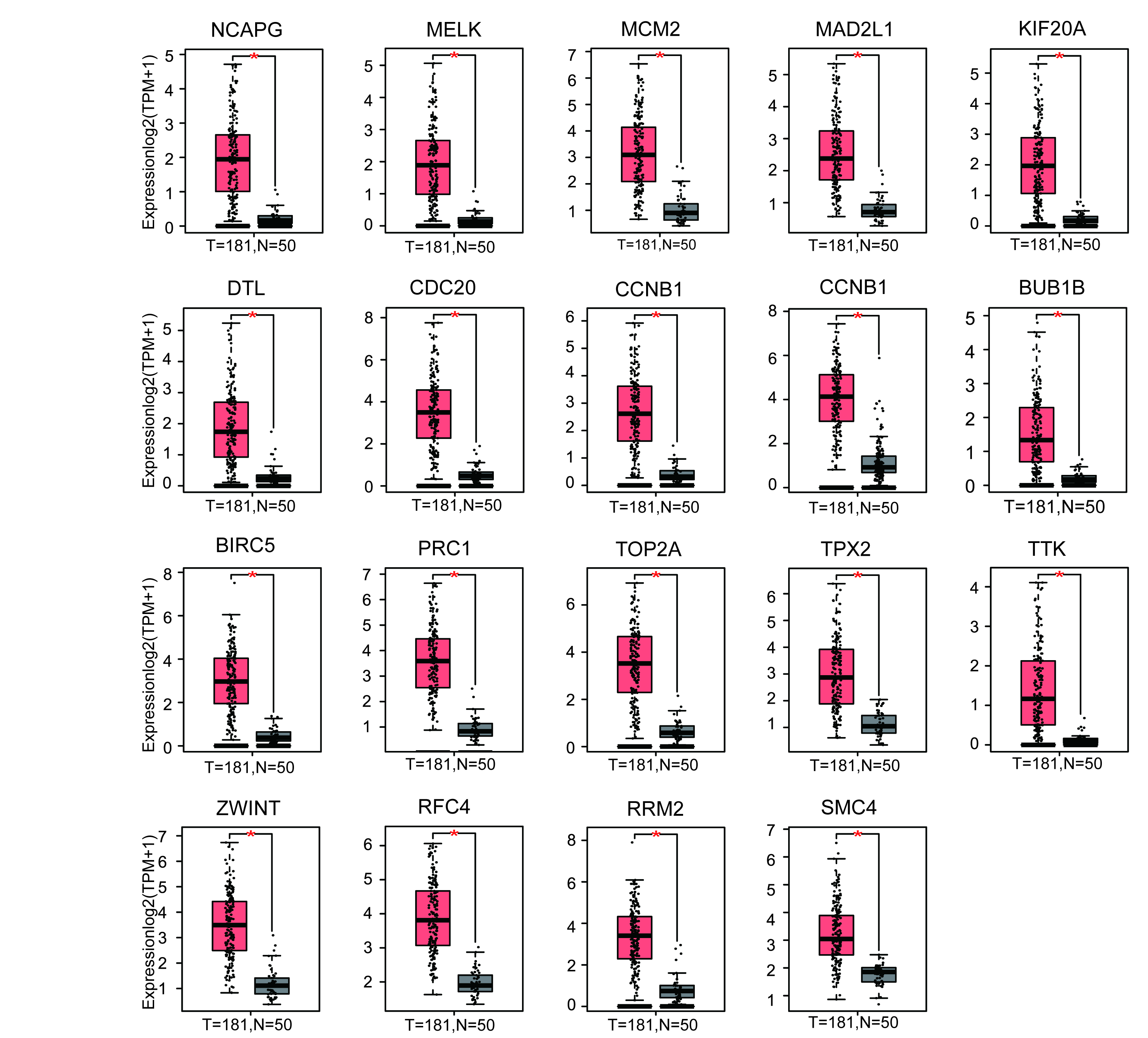

Supplement: Supplementary Figure 5 — External validation of hub genes in the brown module. The hub genes from the brown module expression differences between HCC and adjacent normal tissues in GPEIA2. “∗” represents P value l < 0.05. [file Image_5.TIF]

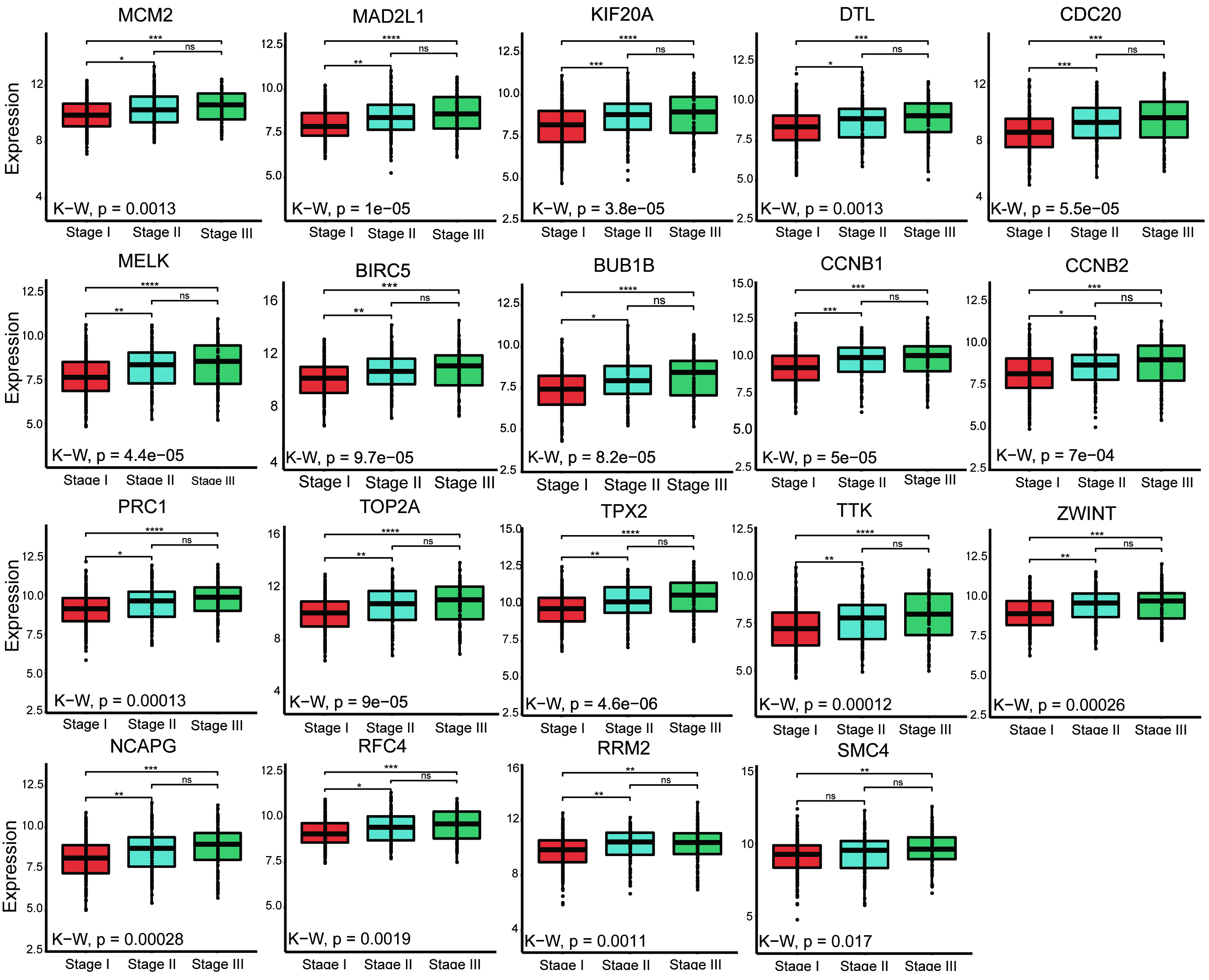

Supplement: Supplementary Figure 6 — External validation of hub genes in the brown module. Expression of these genes in HCC samples with different TNN stages. “∗” represents P value < 0.05; “∗∗” represents P value l < 0.01; “∗∗∗” represents P value < 0.001. [file Image_6.TIF]

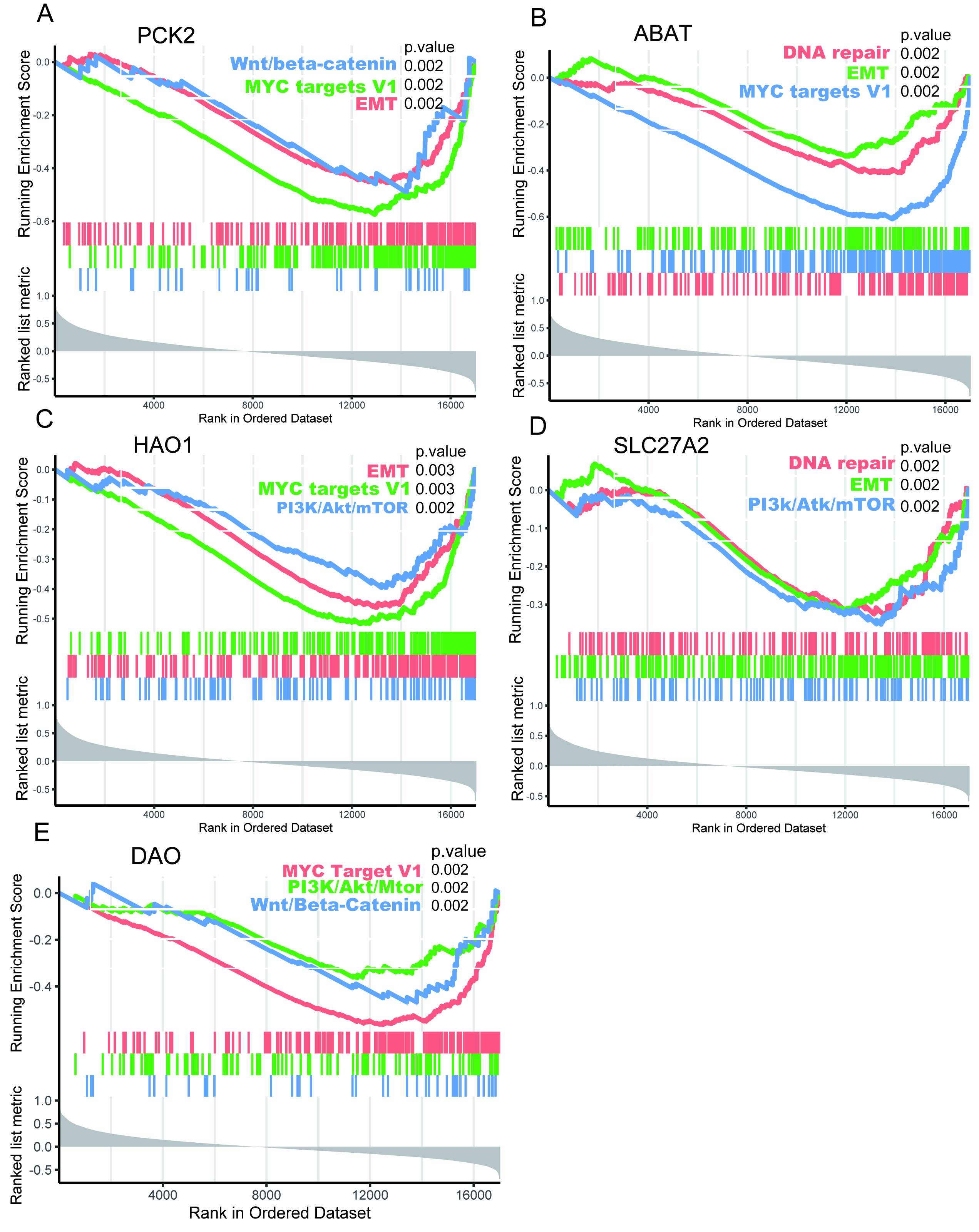

Supplement: Supplementary Figure 7 — Gene sets related to cancer. Results of GSEA related to cancer in samples negatively correlated with PCK2 (A), ABAT (B), HAO1 (C), SLC27A2 (D), and DAO (E) expression. Highlight 3 gene sets for each gene. [file Image_7.TIF]

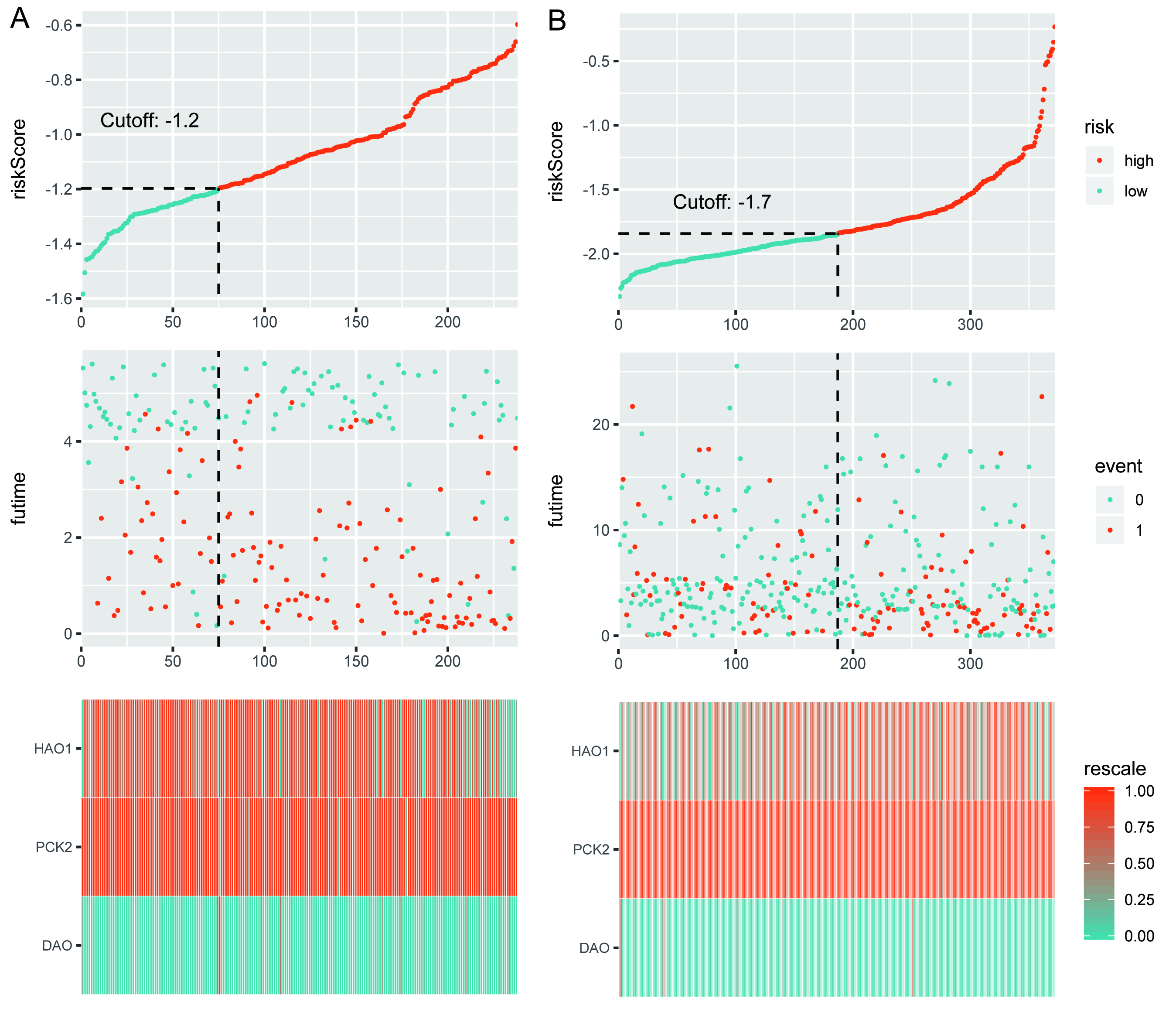

Supplement: Supplementary Figure 8 — Risk score distribution, survival status, and heatmaps for patients in the GSE14520 (A) and TCGA-LIHC (B) datasets divided into high- and low-risk groups. [file Image_8.TIF]

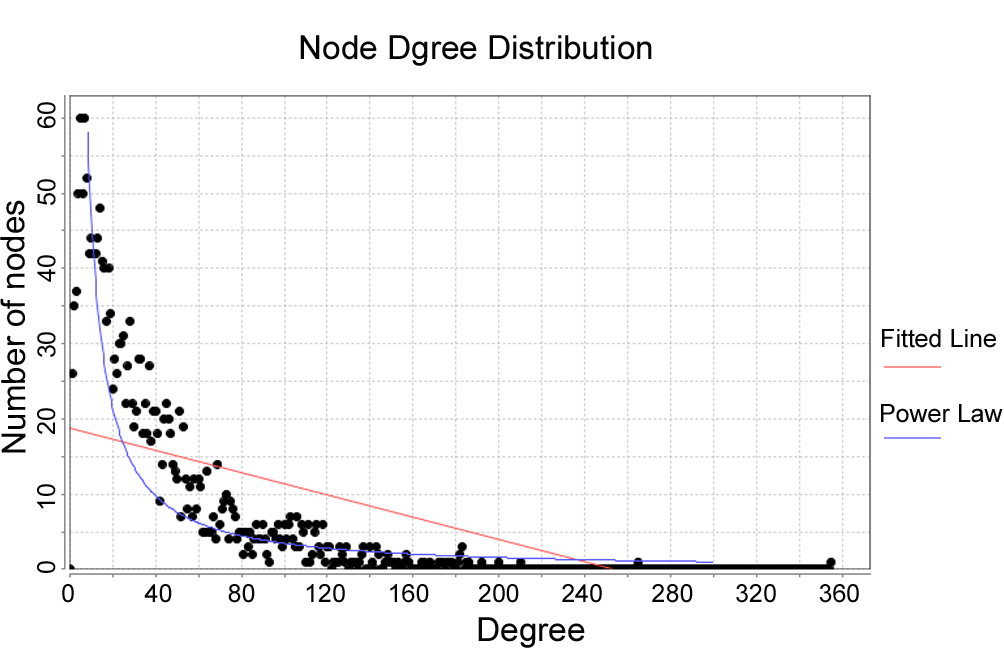

Supplement: Supplementary Figure 9 — Node degree distribution plot of differentially expressed genes. [file Image_9.TIF]
