# Supplementary material for: Identification of Hub Genes Associated With Hepatocellular Carcinoma Using Robust Rank Aggregation Combined With Weighted Gene Co-expression Network Analysis
Source: Front Genet. 2020 Sep 30;11:895. doi: 10.3389/fgene.2020.00895 (PMC7561391; doi:10.3389/fgene.2020.00895)
Supplement: Supplementary Table 1 — BP of GO analysis for brown module. [file Table_1.DOCX]

Supplementary Table 1 BP of GO analysis for brown module.

| **ID** | **Description** | **p.adjust** | **Count** |
| --- | --- | --- | --- |
| GO:0006260 | DNA replication | 3.78E-35 | 60 |
| GO:0006261 | DNA-dependent DNA replication | 7.47E-27 | 39 |
| GO:0000819 | sister chromatid segregation | 1.11E-23 | 39 |
| GO:1901987 | regulation of cell cycle phase transition | 1.11E-23 | 63 |
| GO:0140014 | mitotic nuclear division | 1.69E-23 | 46 |
| GO:0000070 | mitotic sister chromatid segregation | 1.69E-23 | 36 |
| GO:0007059 | chromosome segregation | 1.69E-23 | 49 |
| GO:1901990 | regulation of mitotic cell cycle phase transition | 1.76E-23 | 60 |
| GO:0044843 | cell cycle G1/S phase transition | 9.26E-22 | 48 |
| GO:0000082 | G1/S transition of mitotic cell cycle | 2.61E-21 | 46 |
| GO:0098813 | nuclear chromosome segregation | 2.75E-21 | 42 |
| GO:0000280 | nuclear division | 4.97E-21 | 52 |
| GO:0010948 | negative regulation of cell cycle process | 5.53E-21 | 51 |
| GO:0048285 | organelle fission | 7.84E-20 | 53 |
| GO:0045930 | negative regulation of mitotic cell cycle | 8.27E-20 | 47 |
| GO:0033260 | nuclear DNA replication | 1.30E-18 | 21 |
| GO:0044786 | cell cycle DNA replication | 6.54E-18 | 22 |
| GO:0044839 | cell cycle G2/M phase transition | 6.54E-18 | 41 |
| GO:0007051 | spindle organization | 6.54E-18 | 32 |

BP,biological process;GO, Gene Ontology
