# Supplementary material for: Identification of Hub Genes Associated With Hepatocellular Carcinoma Using Robust Rank Aggregation Combined With Weighted Gene Co-expression Network Analysis
Source: Front Genet. 2020 Sep 30;11:895. doi: 10.3389/fgene.2020.00895 (PMC7561391; doi:10.3389/fgene.2020.00895)
Supplement: Supplementary Table 2 — CC of GO analysis for brown module. [file Table_2.DOCX]

Supplementary Table 2 CC of GO analysis for brown module.

| **ID** | **Description** | **p.adjust** | **Count** |
| --- | --- | --- | --- |
| GO:0098687 | chromosomal region | 2.55E-27 | 57 |
| GO:0000793 | condensed chromosome | 7.28E-24 | 42 |
| GO:0000775 | chromosome, centromeric region | 3.54E-21 | 38 |
| GO:0000779 | condensed chromosome, centromeric region | 2.98E-19 | 29 |
| GO:0000776 | kinetochore | 3.21E-19 | 31 |
| GO:0000777 | condensed chromosome kinetochore | 2.47E-18 | 27 |
| GO:0005819 | spindle | 2.73E-18 | 45 |
| GO:0044454 | nuclear chromosome part | 3.04E-14 | 48 |
| GO:0000922 | spindle pole | 8.44E-11 | 24 |
| GO:0000785 | chromatin | 8.97E-11 | 44 |
| GO:0072686 | mitotic spindle | 3.10E-10 | 19 |
| GO:0005657 | replication fork | 3.34E-09 | 14 |
| GO:0005635 | nuclear envelope | 4.38E-09 | 37 |
| GO:0034399 | nuclear periphery | 2.64E-08 | 19 |
| GO:0030496 | midbody | 2.64E-08 | 22 |
| GO:0000781 | chromosome, telomeric region | 9.13E-07 | 19 |
| GO:0000790 | nuclear chromatin | 5.55E-06 | 26 |
| GO:0018995 | host | 6.06E-06 | 12 |
| GO:0043657 | host cell | 6.06E-06 | 12 |
| GO:0005874 | microtubule | 6.78E-06 | 28 |

CC, cellular component; GO, Gene Ontology
