# Supplementary material for: Identification of Hub Genes Associated With Hepatocellular Carcinoma Using Robust Rank Aggregation Combined With Weighted Gene Co-expression Network Analysis
Source: Front Genet. 2020 Sep 30;11:895. doi: 10.3389/fgene.2020.00895 (PMC7561391; doi:10.3389/fgene.2020.00895)
Supplement: Supplementary Table 3 — MF of GO analysis for brown module. [file Table_3.DOCX]

Supplementary Table 3 MF of GO analysis for brown module.

| **ID** | **Description** | **P.adjust** | **Count** |
| --- | --- | --- | --- |
| GO:0140097 | catalytic activity, acting on DNA | 6.84E-09 | 24 |
| GO:0003684 | damaged DNA binding | 4.05E-07 | 14 |
| GO:0008094 | DNA-dependent ATPase activity | 2.86E-05 | 13 |
| GO:0003697 | single-stranded DNA binding | 4.84E-05 | 14 |
| GO:0004386 | helicase activity | 0.000118 | 16 |
| GO:0003682 | chromatin binding | 0.000146 | 32 |
| GO:0000400 | four-way junction DNA binding | 0.000383 | 6 |
| GO:0016887 | ATPase activity | 0.00057 | 23 |
| GO:0043142 | single-stranded DNA-dependent ATPase activity | 0.001131 | 5 |
| GO:0042623 | ATPase activity, coupled | 0.001298 | 19 |
| GO:0003887 | DNA-directed DNA polymerase activity | 0.001354 | 6 |
| GO:0003689 | DNA clamp loader activity | 0.001354 | 4 |
| GO:0033170 | protein-DNA loading ATPase activity | 0.001354 | 4 |
| GO:0032137 | guanine/thymine mispair binding | 0.001362 | 3 |
| GO:0034061 | DNA polymerase activity | 0.001448 | 7 |
| GO:0017056 | structural constituent of nuclear pore | 0.001534 | 5 |
| GO:0000217 | DNA secondary structure binding | 0.001677 | 6 |
| GO:0004674 | protein serine/threonine kinase activity | 0.002933 | 25 |
| GO:0004523 | RNA-DNA hybrid ribonuclease activity | 0.004931 | 4 |
| GO:0030983 | mismatched DNA binding | 0.004931 | 4 |

MF, molecular function; GO, Gene Ontology
