# Supplementary material for: Identification of Hub Genes Associated With Hepatocellular Carcinoma Using Robust Rank Aggregation Combined With Weighted Gene Co-expression Network Analysis
Source: Front Genet. 2020 Sep 30;11:895. doi: 10.3389/fgene.2020.00895 (PMC7561391; doi:10.3389/fgene.2020.00895)
Supplement: Supplementary Table 4 — BP of GO analysis for turquoise module. [file Table_4.DOCX]

Supplementary Table 4 BP of GO analysis for turquoise module.

| **ID** | **Description** | **p.adjust** | **Count** |
| --- | --- | --- | --- |
| GO:0044282 | small molecule catabolic process | 6.67E-46 | 121 |
| GO:0016054 | organic acid catabolic process | 5.83E-44 | 93 |
| GO:0046395 | carboxylic acid catabolic process | 5.83E-44 | 93 |
| GO:0006520 | cellular amino acid metabolic process | 4.76E-33 | 96 |
| GO:1901605 | alpha-amino acid metabolic process | 9.48E-30 | 69 |
| GO:0016053 | organic acid biosynthetic process | 4.31E-28 | 100 |
| GO:0046394 | carboxylic acid biosynthetic process | 1.46E-27 | 99 |
| GO:0008202 | steroid metabolic process | 5.43E-27 | 84 |
| GO:0006631 | fatty acid metabolic process | 6.04E-26 | 85 |
| GO:0009063 | cellular amino acid catabolic process | 9.30E-26 | 49 |
| GO:0006732 | coenzyme metabolic process | 1.39E-25 | 88 |
| GO:0044242 | cellular lipid catabolic process | 4.70E-22 | 58 |
| GO:1901615 | organic hydroxy compound metabolic process | 4.95E-22 | 99 |
| GO:0072329 | monocarboxylic acid catabolic process | 8.58E-22 | 45 |
| GO:1901606 | alpha-amino acid catabolic process | 4.31E-21 | 41 |
| GO:0006805 | xenobiotic metabolic process | 8.50E-21 | 43 |
| GO:0016042 | lipid catabolic process | 1.00E-20 | 72 |
| GO:0009410 | response to xenobiotic stimulus | 2.32E-19 | 66 |
| GO:0071466 | cellular response to xenobiotic stimulus | 7.44E-19 | 50 |
| GO:0072330 | monocarboxylic acid biosynthetic process | 1.51E-18 | 71 |

BP, biological process; GO, Gene Ontology
