# Supplementary material for: Identification of Hub Genes Associated With Hepatocellular Carcinoma Using Robust Rank Aggregation Combined With Weighted Gene Co-expression Network Analysis
Source: Front Genet. 2020 Sep 30;11:895. doi: 10.3389/fgene.2020.00895 (PMC7561391; doi:10.3389/fgene.2020.00895)
Supplement: Supplementary Table 5 — CC of GO analysis for turquoise module. [file Table_5.DOCX]

Supplementary Table 5 CC of GO analysis for turquoise module.

| **ID** | **Description** | **p.adjust** | **Count** |
| --- | --- | --- | --- |
| GO:0005759 | mitochondrial matrix | 7.30E-16 | 81 |
| GO:0031983 | vesicle lumen | 3.66E-15 | 67 |
| GO:0060205 | cytoplasmic vesicle lumen | 8.22E-15 | 66 |
| GO:0072562 | blood microparticle | 1.79E-14 | 41 |
| GO:0034774 | secretory granule lumen | 7.99E-14 | 62 |
| GO:0005777 | peroxisome | 1.35E-13 | 37 |
| GO:0042579 | microbody | 1.35E-13 | 37 |
| GO:0044438 | microbody part | 1.35E-13 | 32 |
| GO:0044439 | peroxisomal part | 1.35E-13 | 32 |
| GO:0005782 | peroxisomal matrix | 4.55E-11 | 21 |
| GO:0031907 | microbody lumen | 4.55E-11 | 21 |
| GO:0034358 | plasma lipoprotein particle | 9.27E-09 | 16 |
| GO:1990777 | lipoprotein particle | 9.27E-09 | 16 |
| GO:0032994 | protein-lipid complex | 2.24E-08 | 16 |
| GO:0034364 | high-density lipoprotein particle | 4.68E-08 | 13 |
| GO:0034709 | methylosome | 4.26E-06 | 8 |
| GO:0030055 | cell-substrate junction | 7.00E-06 | 53 |
| GO:0005925 | focal adhesion | 9.12E-06 | 52 |
| GO:0005924 | cell-substrate adherens junction | 1.01E-05 | 52 |
| GO:0005778 | peroxisomal membrane | 1.05E-05 | 16 |

CC, cellular component; GO, Gene Ontology
