# Supplementary material for: Identification of Hub Genes Associated With Hepatocellular Carcinoma Using Robust Rank Aggregation Combined With Weighted Gene Co-expression Network Analysis
Source: Front Genet. 2020 Sep 30;11:895. doi: 10.3389/fgene.2020.00895 (PMC7561391; doi:10.3389/fgene.2020.00895)
Supplement: Supplementary Table 6 — MF of GO analysis for turquoise module. [file Table_6.DOCX]

Supplementary Table 6 MF of GO analysis for turquoise module.

| **ID** | **Description** | **p.adjust** | **Count** |
| --- | --- | --- | --- |
| GO:0048037 | cofactor binding | 3.69E-32 | 110 |
| GO:0050662 | coenzyme binding | 5.91E-26 | 75 |
| GO:0016614 | oxidoreductase activity, acting on CH-OH group of donors | 7.44E-10 | 33 |
| GO:1901681 | sulfur compound binding | 1.94E-08 | 44 |
| GO:0004497 | monooxygenase activity | 6.46E-08 | 25 |
| GO:0016616 | oxidoreductase activity, acting on the CH-OH group of donors, NAD or NADP as acceptor | 8.52E-08 | 28 |
| GO:0008514 | organic anion transmembrane transporter activity | 1.10E-07 | 32 |
| GO:0016627 | oxidoreductase activity, acting on the CH-CH group of donors | 1.89E-07 | 18 |
| GO:0019842 | vitamin binding | 1.89E-07 | 30 |
| GO:0005506 | iron ion binding | 2.00E-07 | 32 |
| GO:0016903 | oxidoreductase activity, acting on the aldehyde or oxo group of donors | 1.85E-06 | 15 |
| GO:0016874 | ligase activity | 1.88E-06 | 29 |
| GO:0005496 | steroid binding | 1.99E-06 | 22 |
| GO:0031406 | carboxylic acid binding | 1.99E-06 | 34 |
| GO:0043177 | organic acid binding | 2.49E-06 | 34 |
| GO:0050660 | flavin adenine dinucleotide binding | 4.82E-06 | 19 |
| GO:0009055 | electron transfer activity | 7.13E-06 | 23 |
| GO:0046943 | carboxylic acid transmembrane transporter activity | 8.25E-06 | 23 |
| GO:0020037 | heme binding | 8.55E-06 | 24 |
| GO:0005342 | organic acid transmembrane transporter activity | 9.07E-06 | 23 |

MF, molecular function; GO, Gene Ontology
