# Supplementary material for: Identification of Hub Genes Associated With Hepatocellular Carcinoma Using Robust Rank Aggregation Combined With Weighted Gene Co-expression Network Analysis
Source: Front Genet. 2020 Sep 30;11:895. doi: 10.3389/fgene.2020.00895 (PMC7561391; doi:10.3389/fgene.2020.00895)
Supplement: Supplementary Table 7 — Enriched function of the miRNA-mRNA network by miRNet. [file Table_7.DOCX]

Supplementary Table S7 Enrich function of miRNA-mRNA network by miRNet

| **Pathway** | **Pval** |
| --- | --- |
| p53 signaling pathway | 0.00075 |
| Propanoate metabolism | 0.00159 |
| Butanoate metabolism | 0.00206 |
| beta-Alanine metabolism | 0.00223 |
| Oocyte meiosis | 0.00289 |
| Cell cycle | 0.00428 |
| Valine leucine and isoleucine degradation | 0.00631 |
| Complement and coagulation cascades | 0.0142 |
