# Supplementary material for: Identification of Hub Genes Associated With Hepatocellular Carcinoma Using Robust Rank Aggregation Combined With Weighted Gene Co-expression Network Analysis
Source: Front Genet. 2020 Sep 30;11:895. doi: 10.3389/fgene.2020.00895 (PMC7561391; doi:10.3389/fgene.2020.00895)
Supplement: Supplementary Table 8 — Survival analysis for the rest of the hub genes. [file Table_8.DOCX]

Supplementary Table S8 survival analysis for the rest of real hub genes.

| **Gene Symbol** | **HR** | **95% CI** | **p.value** |
| --- | --- | --- | --- |
| CCNB1 | 1.4 | (1.2-1.6) | 1.60E-05 |
| TOP2A | 1.2 | (1.1-1.4) | 0.001 |
| RFC4 | 1.4 | (1.1-1.7) | 9.00E-04 |
| MAD2L1 | 1.3 | (1.1-1.6) | 0.00036 |
| BUB1B | 1.3 | (1.1-1.4) | 0.00027 |
| CDC20 | 1.3 | (1.2-1.5) | 1.50E-06 |
| CCNB2 | 1.2 | (1.1-1.4) | 0.0049 |
| BIRC5 | 1.2 | (1.1-1.4) | 0.00019 |
| RRM2 | 1.3 | (1.1-1.5) | 0.00066 |
| TTK | 1.3 | (1.2-1.5) | 1.70E-05 |
| NCAPG | 1.3 | (1.2-1.5) | 7.40E-05 |
| MCM2 | 1.4 | (1.2-1.6) | 0.00012 |
| MELK | 1.3 | (1.1-1.5) | 8.80E-05 |
| PRC1 | 1.2 | (1.1-1.4) | 0.0028 |
| ZWINT | 1.3 | (1.1-1.6) | 0.00024 |
| SMC4 | 1.2 | (1.1-1.4) | 0.0037 |
| KIF20A | 1.4 | (1.2-1.6) | 3.80E-06 |
| DTL | 1.2 | (1-1.4) | 0.0089 |
| TPX2 | 1.4 | (1.2-1.7) | 3.20E-06 |
| CAT | 0.78 | (0.67-0.91) | 0.0019 |
| PCK2 | 0.86 | (0.77-0.96) | 0.0072 |
| F13B | 0.91 | (0.86-0.97) | 0.0022 |
| EHHADH | 0.85 | (0.77-0.94) | 0.0011 |
| SERPINC1 | 0.92 | (0.87-0.97) | 0.0022 |

Note: HR, hazard ratio; CI, confidence interval
