# Supplementary material for: Identification of Hub Genes Associated With Hepatocellular Carcinoma Using Robust Rank Aggregation Combined With Weighted Gene Co-expression Network Analysis
Source: Front Genet. 2020 Sep 30;11:895. doi: 10.3389/fgene.2020.00895 (PMC7561391; doi:10.3389/fgene.2020.00895)
Supplement: Supplementary Table 9 — Biological functions of the hub genes in HCC. [file Table_9.docx]

**Supplementary table S9 Biological functions of the hub genes in HCC.**

| **Genes** | **Biological functions in HCC** | **Reference** |
| --- | --- | --- |
| CCNB1 | A key gene for identifying early HCC | Wu, et al.,2019 |
| TOP2A | Be regarded as a biomarker in HBV-related HCC | Liao, et al.,2019 |
| RFC4 | Promotes cell proliferation of HepG2 | Arai, et al.,2009 |
| MAD2L1 | Promotes HepG2 cell proliferation, migration and invasion | Arai, et al., 2009 |
| BUB1B | Related to poor prognosis | Li, et al., 2017 |
| CDC20 | Promotes cell proliferation in G2/M-phase | Yang, et al.,2019 |
| CCNB2 | Promotes cell proliferation and migration; inhibits cell apoptosis, and caused S phase arrest in HCC cells | Li, et al.,2014 |
| BIRC5 | Mediates cancer cell survival and tumor maintenance | Li, et al.,2019 |
| RRM2 | Associated with the anticancer activity of sorafenib | Cao, et al.,2013 |
| TTK | Activates Akt and promotes proliferation and migration of HCC cells. | Yang, et al.,2020 |
| NCAPG | Promotes the proliferation of HCC through PI3K/AKT Signaling. | Liu, et al.,2015 |
| MCM2 | Promotes of the HepG2 cell cycle and proliferation  through the cyclin D-dependent kinases 2/7 pathway | Gong, et al.,2019 |
| MELK | An oncogenic kinase essential for early HCC recurrence. | Yang, et al.,2018 |
| PRC1 | Promotes early recurrence of HCC throught the Wnt/β-catenin signalling pathway. | Xia, et al.,2016 |
| ZWINT | Promotes the proliferation of HCC by regulating cell-cycle-related proteins. | Chen, et al.,2016 |
| SMC4 | Associated with tumor de-differentiation,advanced stage and vascular invasion of HCC. | Ying, et al.,2018 |
| KIF20A | Contributes to HCC aggressiveness in patients | Zhou, et al.,2012 |
| DTL | Promotes liver cancer cell growth, decreased senescence, and increase tumorigenesis | Gasnereau, et al.,2012 |
| TPX2 | Correlates with HCC cell proliferation, apoptosis, and EMT. | Chen, et al.,2018 |
| CAT | A Potential biomarker for HCC | Liang, et al.,2015 |
| EHHADH | A biomarker in the occurrence of HCC. | Yang, et al.,2005 |
| SERPINC1 | Serum markers for AFP-negative patients with HCC | Arai, et al., 2009 |
| GYS2 | Negative feedback regulates p53 to limit tumor growth in HBV-related hepatocellular carcinoma. | Li, et al., 2017 |

**Note:** References of Supplementary table S9

Wu, M., Liu, Z., Li, X., Zhang, A., Lin, D., and Li, N. (2019). Analysis of potential key genes in very early hepatocellular carcinoma. World J. Surg. Oncol. 17:77. doi: 10.1186/s12957-019-1616-6

Liao, X., Yu, T., Yang, C., Huang, K., Wang, X., Han, C., et al. (2019). Comprehensive investigation of key biomarkers and pathways in hepatitis B virus-related hepatocellular carcinoma. J. Cancer 10, 5689–5704. doi: 10.7150/jca.31287

Arai, M., Kondoh, N., Imazeki, N., Hada, A., Hatsuse, K., Matsubara, O., et al.(2009). The knockdown of endogenous replication factor C4 decreases the growth and enhances the chemosensitivity of hepatocellular carcinoma cells.Liver Int. 29, 55–62. doi: 10.1111/j.1478-3231.2008.01792.x

Li, Y., Bai, W., and Zhang, J. (2017). MiR-200c-5p suppresses proliferation and metastasis of human hepatocellular carcinoma (HCC) via suppressing MAD2L Li, J., Gao, J. Z., Du, J. L., Huang, Z. X., and Wei, L. X. (2014). Increased CDC20 expression is associated with development and progression of hepatocellular carcinoma. Int. J. Oncol. 45, 1547–1555. doi: 10.3892/ijo.2014.2559

Li, R., Jiang, X., Zhang, Y., Wang, S., Chen, X., Yu, X., et al. (2019). Cyclin B2 overexpression in human hepatocellular carcinoma is associated with poor prognosis. Arch. Med. Res. 50, 10–17. doi: 10.1016/j.arcmed.2019.

03.003

Cao, L., Li, C., Shen, S., Yan, Y., Ji, W., Wang, J., et al. (2013). OCT4 increases BIRC5and CCND1 expression and promotes cancer progression in hepatocellular carcinoma. BMC Cancer 13:82. doi: 10.1186/1471-2407-13-82

Yang, P. M., Lin, L. S., and Liu, T. P. (2020). Sorafenib Inhibits ribonucleotide reductase regulatory subunit M2 (RRM2) in hepatocellular carcinoma cells. Biomolecules 10:117. doi: 10.3390/biom10010117

Liu, X., Liao, W., Yuan, Q., Ou, Y., and Huang, J. (2015). TTK activates Akt and promotes proliferation and migration of hepatocellular carcinoma cells. Oncotarget 6, 34309–34320. doi: 10.18632/oncotarget.5295

Gong, C., Ai, J., Fan, Y., Gao, J., Liu, W., Feng, Q., et al. (2019). NCAPG promotes the proliferation of hepatocellular carcinoma through PI3K/AKT signaling.Onco. Targets Ther. 12, 8537–8552. doi: 10.2147/OTT.S217916

Yang, J., Xie, Q., Zhou, H., Chang, L., Wei, W., Wang, Y., et al. (2018).Proteomic analysis and NIR-II imaging of MCM2 protein in hepatocellular carcinoma. J. Proteome Res. 17, 2428–2439. doi: 10.1021/acs.jproteome.8b00181

Xia, H., Kong, S. N., Chen, J., Shi, M., Sekar, K., Seshachalam, V. P., et al.(2016). MELK is an oncogenic kinase essential for early hepatocellularcarcinoma recurrence. Cancer Lett. 383, 85–93. doi: 10.1016/j.canlet.2016.09.017

Chen, J., Rajasekaran, M., Xia, H., Zhang, X., Kong, S. N., Sekar, K., et al.(2016). The microtubule-associated protein PRC1 promotes early recurrence of hepatocellular carcinoma in association with the Wnt/b-catenin signaling pathway. Gut 65, 1522–1534. doi: 10.1136/gutjnl-2015-310625

Ying, H., Xu, Z., Chen, M., Zhou, S., Liang, X., and Cai, X. (2018). Overexpression of Zwint predicts poor prognosis and promotes the proliferation of hepatocellular carcinoma by regulating cell-cycle-related proteins. Onco.Targets Ther. 11, 689–702. doi: 10.2147/OTT.S152138

Zhou, B., Yuan, T., Liu, M., Liu, H., Xie, J., Shen, Y., et al. (2012). Overexpression of the structural maintenance of chromosome 4 protein is associated with tumor de-differentiation, advanced stage and vascular invasion of primary liver cancer.

Oncol. Rep. 28, 1263–1268. doi: 10.3892/or.2012.1929 Gasnereau, I., Boissan, M.,Margall-Ducos, G., Couchy, G., Wendum, D., Bourgain-

Guglielmetti, F., et al. (2012). KIF20A mRNA and its product MKlp2 are increased during hepatocyte proliferation and hepatocarcinogenesis. Am. J.Pathol. 180, 131–140. doi: 10.1016/j.ajpath.2011.09.040

Chen, Y. C., Chen, I. S., Huang, G. J., Kang, C. H., Wang, K. C., Tsao, M. J.,et al. (2018). Targeting DTL induces cell cycle arrest and senescence and suppresses cell growth and colony formation through TPX2 inhibition in human hepatocellular carcinoma cells. Onco. Targets Ther. 11, 1601–1616. doi:10.2147/OTT.S147453

Liang, B., Jia, C., Huang, Y., He, H., Li, J., Liao, H., et al. (2015). TPX2 level correlates with hepatocellular carcinoma cell proliferation, apoptosis, and EMT.Dig. Dis. Sci. 60, 2360–2372. doi: 10.1007/s10620-015-3730-9

Yang, L. Y., Chen, W. L., Lin, J. W., Lee, S. F., Lee, C. C., Hung, T. I., et al.(2005). Differential expression of antioxidant enzymes in various hepatocellular carcinoma cell lines. J. Cell. Biochem. 96, 622–631. doi: 10.1002/jcb.20541

Arai, M., Kondoh, N., Imazeki, N., Hada, A., Hatsuse, K., Matsubara, O., et al.(2009). The knockdown of endogenous replication factor C4 decreases thegrowth and enhances the chemosensitivity of hepatocellular carcinoma cells.Liver Int. 29, 55–62. doi: 10.1111/j.1478-3231.2008.01792.x

Li, Y., Bai, W., and Zhang, J. (2017). MiR-200c-5p suppresses proliferation and metastasis of human hepatocellular carcinoma (HCC) via suppressing MAD2L1. Biomed. Pharmacother. 92, 1038–1044. doi: 10.1016/j.biopha.2017.05.092
